# Structural Dispersity as a Determinant of Li-Ion Transport in Ethylene-Oxide-Based Graft Polymer Electrolytes

**DOI:** 10.1021/acs.chemmater.5c03475

**Published:** 2026-02-06

**Authors:** Anna Vigolo, Valeria Vanoli, Luca Laugeni, Carlos Pavón, Rossana Pasquino, Edmondo M. Benetti, Franca Castiglione, Francesca Lorandi

**Affiliations:** † Laboratory for Macromolecular and Organic Chemistry, Department of Chemical Sciences, 9308 University of Padova, Via Marzolo 1, 35131 Padova, Italy; ‡ Department of Chemistry, Materials and Chemical Engineering “G. Natta”, 18981 Politecnico di Milano, Piazza L. Da Vinci 32, 20133 Milano, Italy; § DICMaPI, Università degli Studi di Napoli, Federico II, P. le Tecchio 80, 80125 Napoli, Italy

## Abstract

Graft polymers with oligo­(ethylene glycol) (OEG) side chains and poly­(meth)­acrylate backbones have been commonly studied as polymer electrolytes (PEs) owing to the ability of oligoether segments to coordinate Li^+^ ions. However, when poly­[oligo­(ethylene glycol) methyl ether methacrylate]­s (P­(OEG)­MAs) are synthesized from commercial macromonomers, these are structurally polydisperse, as OEG segments feature a broad distribution of lengths. Herein, we investigate the influence of side-chain heterogeneity on Li-ion transport by comparing structurally polydisperse P­(OEG)­MAs with analogous graft polymers with homogeneous architecture, generated from discrete macromonomer feeds obtained through flash chromatography. Ionic conductivity was found to increase with increasing side-chain dispersity. For structurally polydisperse P­(OEG)­MAs, enhancing side-chain heterogeneity resulted in greater salt dissociation and higher ionic conductivity at relatively high salt contents. These trends are uncorrelated with differences in thermal properties, rheology, and polymer diffusivity, indicating that ion transport is not governed by overall polymer dynamics. Dispersity of side chains thus emerges as a determinant for Li-ion transport in PEs based on P­(OEG)­MAs. However, this effect is lost when backbone flexibility increases, i.e., when polymethacrylates are substituted with more flexible polyacrylate counterparts. By elucidating how side-chain heterogeneity and backbone flexibility affect ion transport, this work provides guidance for the rational design of graft PEs.

## Graphical abstract

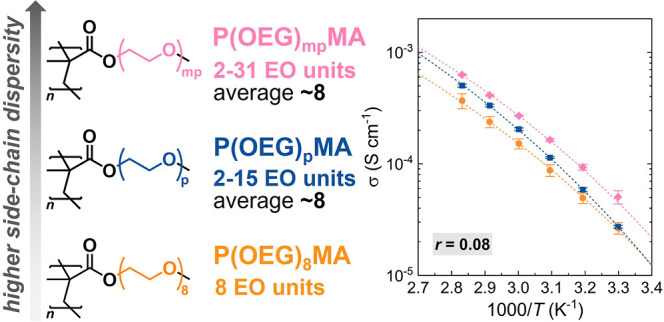

## 1 Introduction

Polymer electrolytes (PEs) are key materials for high-energy-density electrochemical energy storage. For decades, polyethers—and especially poly­(ethylene oxide) (PEO)—have attracted continued interest due to their superior ability to solvate and transport alkali-metal cations. However, the inherent limitations of PEO—primarily arising from its semicrystalline nature—have prompted efforts to improve the electrochemical performance of polyether-based PEs.^1–3^ Several structural parameters, such as end-functionalities and molecular weight, have been extensively studied to elucidate their effects on ion transport.

Dispersity of polymer molar mass (*D̵*) plays a critical role in various properties of polymeric materials, including viscosity, processability, and thermal and mechanical behavior.^4–7^ While dispersity has been exploited to tailor polymer self-assembly in bulk and in solution and tune the mechanical properties of networks and elastomeric materials,^8^ its impact on ion transport in PEs has been rarely considered. Mahanthappa et al. synthesized a series of polystyrene­(PS)-*b*-PEO*-b*-PS triblock copolymers with different *D̵* of the PEO block.^9,10^ They measured an enhancement in ionic conductivity with increasing *D̵*, which was attributed to smaller lamellar grain size in copolymers with more disordered PEO blocks, resulting in improved intergrain connectivity and ion transport. Watanabe et al. compared the ionic conductivities and mechanical properties of various network polymer electrolytes including a homogeneous model network composed of 4-arm poly­(ethylene glycol) (tetra-PEG).^11,12^ They found that the average network size played a more significant role than the mesh size distribution in governing ion transport, although the homogeneous network exhibited superior toughness.

Graft polymers bearing oligo­(ethylene glycol) (OEG) side chains hold promise as components of PEs.^1,13–16^ Their relatively short oligoether side chains provide polymers with reduced crystallinity compared with PEO, resulting in enhanced ionic conductivity at lower temperatures. Moreover, the design space of macromonomers with OEG side chains is vast.^17–23^ Their polymerization can be achieved through various techniques, including ionic polymerizations and reversible deactivation radical polymerizations, enabling control over (co)­polymer architecture and composition.^13,24^ A common—yet often overlooked—feature of these macromonomers is that they typically present a distribution of OEG side-chain lengths rather than a discrete number of ethylene oxide (EO) units. The extent to which this intrinsic structural dispersity influences ion transport remains unexplored.

Recent works have shown that the dispersity of OEG side chains can serve as a synthetic tool to manipulate various polymer properties.^25–29^ Lawrence et al. reported that uniform bottlebrushes featuring discrete backbone and OEG side chains and a fluorinated terminus outperform their heterogeneous counterparts as ^19^F MRI contrast agents.^25^ Our group demonstrated that poly­[oligo­(ethylene glycol) methyl ether methacrylate] (P­(OEG)­MA) brushes comprising uniform OEG side chains exhibit increased hydration, lubricity, and colloidal stability in comparison with parent brushes bearing a distribution of side-chain lengths.^26,27^

Aiming to assess the influence of structural dispersity on Li-ion transport, we synthesized poly­(meth)­acrylates with discrete and variably disperse OEG side chains and blended them with lithium bis­(trifluoromethanesulfonyl)­imide (LiTFSI). The thermal and rheological properties, polymer and ion diffusivity, and conductivity of these PEs were investigated and correlated with the structural features of the polymers.

Previous experimental and theoretical studies on P­(OEG)­MA-based PEs revealed that the length of OEG side chains is the main factor determining Li-ion conductivity.^21,30^ This behavior was attributed to heterogeneous polymer dynamics resulting from the graft architecture. The EO units close to the relatively immobile backbone show slow relaxations, whereas those farther from the backbone participate much more effectively in Li-ion solvation, determining the overall conductivity. In this work, we show that P­(OEG)­MA-based PEs display higher ionic conductivity as the heterogeneity of side-chain lengths increases. This effect becomes less significant for analogous polyacrylates that feature a more flexible backbone. For more rigid polymethacrylate backbones, broadening the distribution of the OEG side-chain lengths enables us to introduce relatively long chains which, on the one hand, increase polymer–polymer and polymer–ion interactions and, on the other hand, improve ion transport. By enhancement of the dispersity of the OEG side chains relative to a commercial (OEG)­MA macromonomer, it is possible to increase the content of Li salt in the corresponding PE while maintaining high salt dissociation and conductivity. Thus, structural dispersity emerges as a complementary design parameter—alongside side-chain length and composition—for optimizing the ion transport properties of graft polymer electrolytes.

## 2 Results and Discussion

Commercially available (OEG)­MA macromonomers are mixtures of methacrylates bearing OEG side chains with varying degrees of polymerization. We focused on the commercial macromonomer with an average molar mass of ∼500 g mol^–1^ ((OEG)_p_MA-500, where “p” stands for “polydisperse” to account for the presence of multiple species). This choice was motivated by several factors: (i) PEs based on (OEG)_p_MA-500 exhibit higher ionic conductivity (σ) than those based on (OEG)_p_MA with *M*_*n*_ ∼ 300 g mol^–1^ ((OEG)_p_MA-300);^21^ (ii) the higher crystallinity^31^ of (OEG)_p_MA with *M*_*n*_ ∼ 950 g mol^–1^ ((OEG)_p_MA-950) compared to (OEG)_p_MA-500 can be detrimental for Li-ion transport; (iii) the entanglement molecular weight of PEO is ∼1–2 kg mol^–1^,^22,32^ making P­(OEG)_p_MA with longer side chains more likely to form entanglements that reduce Li-ion mobility, particularly at room temperature.

A combination of ultraperformance liquid chromatography (UPLC) and high-performance LC coupled with electrospray ionization mass spectrometry (HPLC-ESI/MS) revealed that (OEG)_p_MA-500 featured a distribution of OEG side chains ranging from 2 to 15 EO units (Figures 1 and S1 and Table S1).^26^ This determines a calculated number-average molar mass *M*_*n*,calc_ = 466 ± 117 g mol^–1^ and dispersity *D̵* = 1.06 (Table S2). The most abundant species (14.6 mol %) features 8 EO units in the side chain, i.e., (OEG)_8_MA (molar mass of 452 g mol^–1^). Flash silica gel chromatography was employed to isolate the discrete species (OEG)_8_MA in good yield (∼60%).^26,33^ HPLC-ESI/MS analysis of the discrete macromonomer sample revealed the presence of traces of (OEG)_7_MA and (OEG)_9_MA, while (OEG)_8_MA accounted for 98.5 mol % (Figures 1 and S2 and Tables S3 and S4), resulting in *M*_*n*,calc_ = 452 ± 5 g mol^–1^ and dispersity *D̵* = 1.00014 (hereafter approximated as *D̵* = 1.00). ^1^H NMR spectra of (OEG)_p_MA-500 and (OEG)_8_MA showed an average number of EO units *n*_av,EO_ = 8.7 and 8.2 (Figures S3 and S4).

**Figure 1:**
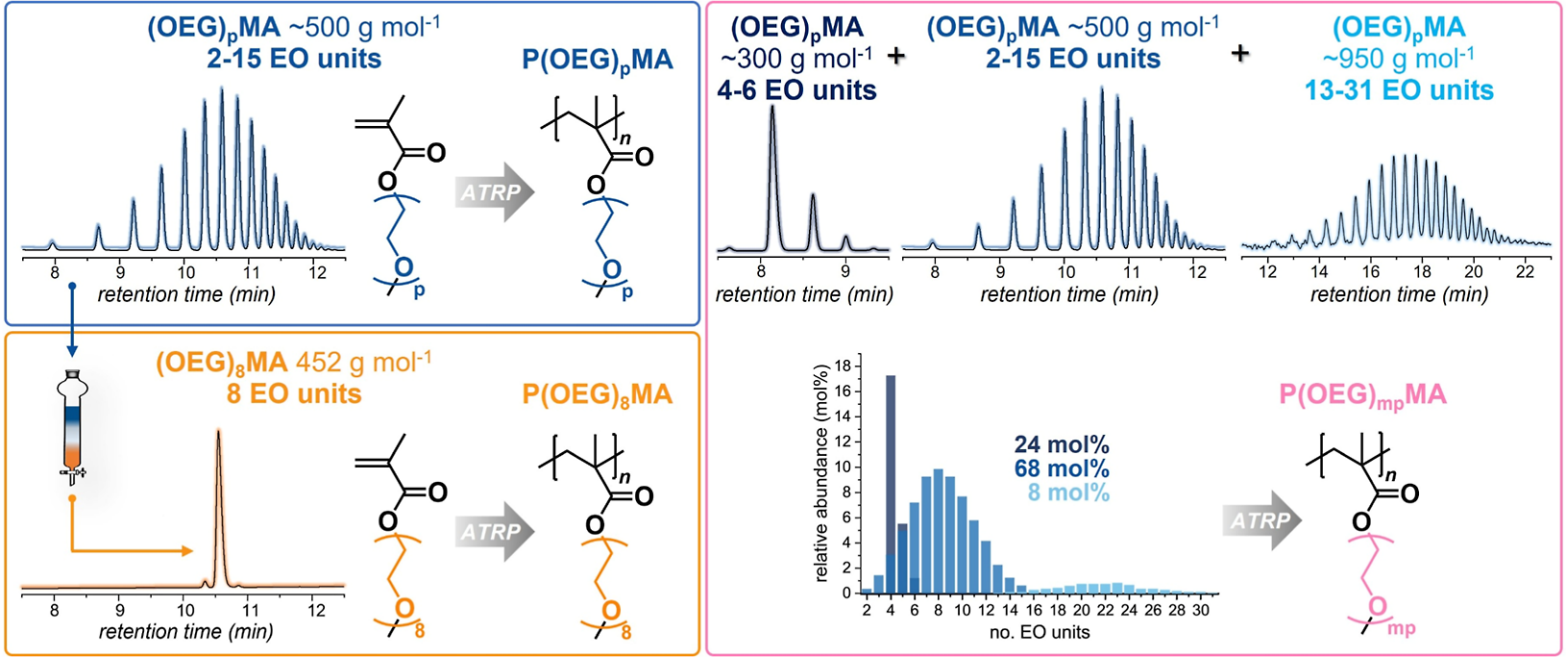
Chemical structure and UPLC elugrams of the commercial macromonomers (OEG)_p_MA-300, (OEG)_p_MA-500, and (OEG)_p_MA-950 (with *M*_*n*_ ∼ 300, ∼500, and ∼950 g mol^–1^) and the discrete (OEG)_8_MA separated by flash chromatography from (OEG)_p_MA-500. Chemical structure of the polymers derived from (OEG)_8_MA, (OEG)_p_MA-500, and the macromonomers’ mix giving a polymer with increased side-chain heterogeneity, i.e., P­(OEG)_mp_MA.

The corresponding polymers with discrete and heterogeneous OEG side chains, P­(OEG)_8_MA and P­(OEG)_p_MA, were prepared by activators regenerated by electron transfer atom transfer radical polymerization (ARGET ATRP, Figures S3–S6). Different batches of the two polymers were prepared under identical polymerization conditions to enable statistically robust characterizations. Purified polymers showed *M*_*n*_ values of 20–29 kg mol^–1^ and main-chain *D̵* = 1.3–1.4 (Figures S9 and S10 and Table S8). ^1^H NMR spectra revealed *n*_av,EO_ = 8.8 and 8.2 for P­(OEG)_p_MA and P­(OEG)_8_MA, respectively (Figures S12 and S13), identical with the corresponding macromonomers.

Additionally, we reasoned that the heterogeneity of the OEG side chains could be intentionally enhanced by mixing (OEG)_p_MA macromonomers with different OEG side-chain distributions to form P­(OEG)_mp_MA, where “mp” stands for “more polydisperse”. Commercial (OEG)_p_MA-300 and (OEG)_p_MA-950 were also analyzed by UPLC and HPLC-ESI/MS (Figure 1 and Table S1). (OEG)_p_MA-300 showed a rather narrow distribution of species, with *M*_*n*,calc_ = 291 ± 25 g mol^–1^ and *D̵* = 1.01. In contrast, (OEG)_p_MA-950 comprised at least 19 different species with 13–31 EO units in the side chain, resulting in *M*_*n*,calc_ = 1056 ± 169 g mol^–1^ and *D̵* = 1.03. Thus, we prepared a macromonomer mixture comprising 24, 68, and 8 mol % of (OEG)_p_MA-300, (OEG)_p_MA-500, and (OEG)_p_MA-950, respectively. The mixture has *M*_*n*,calc_ = 471 ± 217 g mol^–1^ and *n*_av,EO_ = 8.5 (Table S5), comparable with (OEG)_p_MA-500 and (OEG)_8_MA. However, *D̵* = 1.21, which is substantially larger than the dispersity of (OEG)_p_MA-500 (*D̵* = 1.06) and of the discrete (OEG)_8_MA (*D̵* = 1.00).

P­(OEG)_mp_MA was generated by ARGET ATRP of the macromonomer mixture, reaching nearly quantitative conversion to ensure the incorporation of all different (OEG)­MAs into the polymer (Table 1). P­(OEG)_mp_MA samples exhibited *M*_*n*_ = 27 kDa, main-chain *D̵* = 1.3–1.4 (Table S8 and Figure S11), and *n*_av,EO_ = 8.8, according to ^1^H NMR analysis (Figure S14).

**Table 1:** Structural Parameters, *T*_g_, and Ionic Conductivity (σ) of Poly­(meth)­acrylates with Discrete and Disperse OEG Side Chains

polymer	*M*_*n*_ ^a^ (kg mol^–1^)	*D̵* ^a^	*T*_g_ (°C)	*T*_g,*r*=0.08_ (°C)	σ_*r*=0.08,50 °C_ ^b^ (10^4^ S cm^–1^)	σ_*r*=0.05,50 °C_ ^b^ (10^4^ S cm^–1^)	*E*_a_ (kJ mol^–1^)
P(OEG)_8_MA	26.9	1.44	–62	–47	0.88 ± 0.11	1.32 ± 0.06	9.89 ± 0.16
P(OEG)_p_MA	29.1	1.35	–61	–40	1.14 ± 0.02	1.69 ± 0.15	10.07 ± 0.06
P(OEG)_mp_MA	27.3	1.41	–65	–39	1.64 ± 0.06	1.79 ± 0.25	8.78 ± 0.17
P(OEG)_8_A	33.5	1.15	–64	–47	2.49 ± 0.02	3.09	9.44 ± 0.07
P(OEG)_p_A	32.5	1.18	–63	–45	2.53 ± 0.11	2.94	9.04 ± 0.11

a Measured by GPC in DMF + 0.01 M LiBr as eluent, using poly­(methyl methacrylate) standards.

b For polymethacrylate-based electrolytes, conductivity values are reported as the average of measurements on different polymers with similar structural and thermal characteristics (the polymer library is reported in Table S8).

The thermal properties of P­(OEG)_8_MA, P­(OEG)_p_MA, and P­(OEG)_mp_MA were measured by differential scanning calorimetry (DSC, Figure 2) and thermogravimetric analysis (TGA, Figure S24). The polymers showed no substantial difference in glass transition and decomposition temperatures (*T*_g_s of approximately −62 °C, and *T*_d_s ∼ 370 °C, respectively, Tables S9). The polymer with discrete side chains, P­(OEG)_8_MA, exhibited a completely amorphous character, as relatively short side chains prevent the formation of ordered domains.^22,29^ For the polymers with heterogeneous side chains, no crystallization occurred during the cooling stage; however, cold crystallization transitions were detected at *T*_cc_ = −21 and −31 °C for P­(OEG)_p_MA and P­(OEG)_mp_MA, respectively (Figure 2). The cold crystallization phenomenon was previously reported for P­(OEG)­MAs with discrete side chains bearing 9 and 10 EO units, and *T*_cc_ values decreased with increasing the number of EO units.^29^ For P­(OEG)_p_MA, this transition is partially coupled with melting (*T*_m_ = −4 °C), whereas for P­(OEG)_mp_MA, it is more distinct, although both the exothermic and melting peaks (*T*_m_ = 3 °C) are rather broad. While side-chain heterogeneity limits the ordering of polymer chains,^29^ the presence of relatively long side chains in P­(OEG)_mp_MA favors the formation of ordered arrangements.

**Figure 2:**
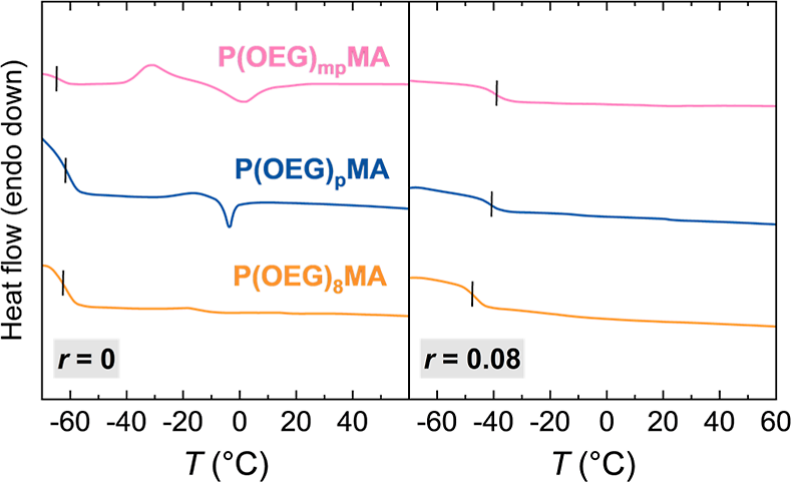
DSC thermograms (heating ramp) of P­(OEG)­MAs without (*r* = 0) and with added LiTFSI (*r* = [Li^+^]/[EO] = 0.08). Curves are vertically offset for clarity. Vertical lines indicate glass transition temperatures.

PEs were prepared by blending each polymer with LiTFSI. The molar ratio of EO units to Li ions was initially fixed at *r* = [Li^+^]/[EO] = 0.08, considering that the highest conductivity of PEO-based electrolytes is typically measured for *r* = 0.05–0.08.^22,34^ The introduction of Li salt resulted in an increase in the *T*_g_ compared to neat polymers (Table 1 and Figure 2). This behavior is typical of PEO-based PEs where associations between ions and polymer segments slows down the segmental relaxations of polymer chains.^20,35^ Additionally, no melting peak was detected for all PEs (Table S9), indicating that the coordination of Li^+^ to ether oxygens disrupts the alignment of OEG side chains in P­(OEG)_p_MA and P­(OEG)_mp_MA, thereby suppressing crystallization.^20,36^ PEs based on P­(OEG)_8_MA showed *T*_g_ values that were 7–8 °C lower than those of PEs with heterogeneous OEG side chains. Previous works highlighted that the addition of Li salt tends to raise *T*_g_ to a greater extent for polymers with shorter OEG side chains, which are more prone to undergo interchain cross-links than intrachain cross-links.^17,20^ Thus, the observed difference in *T*_g_ might originate from the presence of relatively short side chains in P­(OEG)_p_MA and P­(OEG)_mp_MA, which are absent in P­(OEG)_8_MA.

The ionic conductivity (σ) of PEs (*r* = 0.08) was measured by electrochemical impedance spectroscopy (EIS) in a temperature range of 30–80 °C, with 10 °C intervals (Figure 3a). The ionic conductivity increased with an increase in the dispersity of the OEG side chains at nearly all temperatures. PEs based on P­(OEG)_8_MA and P­(OEG)_p_MA showed similar σ values at ambient temperature; however at *T* ≥ 50 °C, the PE with discrete OEG side chains showed consistently lower conductivity. At 50 °C, the σ value of P­(OEG)_8_MA fell below the common benchmark for PEs of 0.1 mS cm^–1^. Within the explored temperature range, PE featuring the broadest distribution of side-chain lengths, P­(OEG)_mp_MA, displayed σ values approximately twice those of P­(OEG)_8_MA and 1.5 times higher than those of P­(OEG)_p_MA (Table 1).

**Figure 3:**
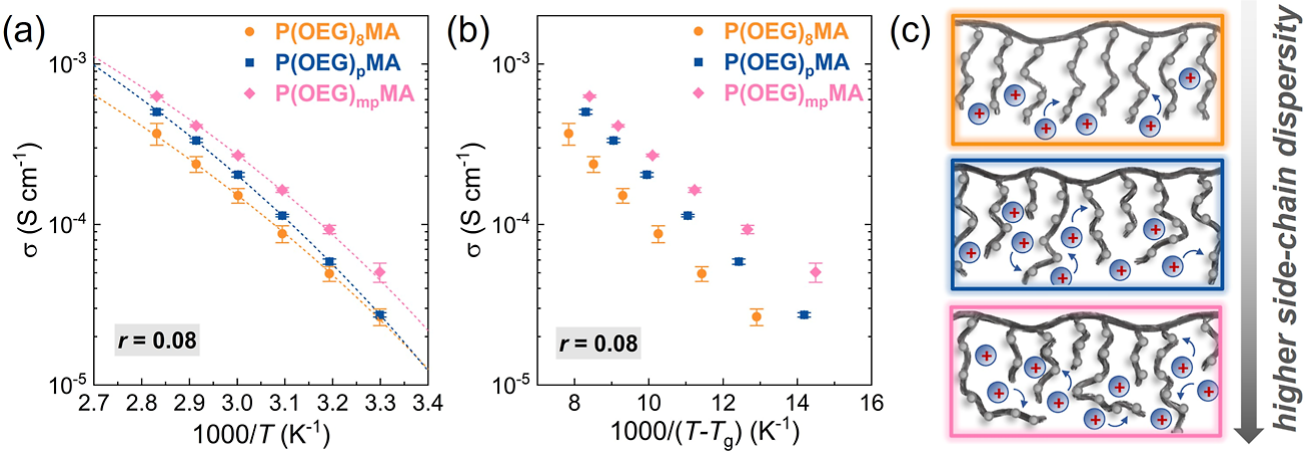
Ionic conductivity (σ) measured by EIS (a) and corrected by *T*_g_ (b) of PEs comprising LiTFSI (*r* = [Li^+^]/[EO] = 0.08) and polymethacrylates with discrete and disperse OEG side chains. In (a), σ values were fitted using the Vogel–Tammann–Fulcher (VTF) equation, where the Vogel temperature *T*_0_ = *T*_g_ – 50 K. (c) Cartoon illustrating the distribution of OEG side chains in the PEs and their interactions with Li ions.

For each P­(OEG)­MA, conductivity measurements were performed on at least three independently prepared samples, using different polymer batches (Table S8) and freshly prepared LiTFSI blends each time. The relatively small error bars reported in Figure 3a indicate that variations in molar mass, main-chain dispersity, and sample preparation have a negligible effect on ion-transport properties, whereas side-chain dispersity is the primary descriptor for these PEs.

Temperature-dependent conductivity data for all PEs were fitted using the Vogel–Tammann–Fulcher (VTF) equation:

1 $$\sigma =Ae^{\frac{-E_{a}}{R\left(T-T_{0}\right)}}$$

where *E*_a_ is the pseudoactivation energy, *A* is a constant prefactor, and *T*_0_ is the Vogel temperature, defined as *T*_0_ = *T*_g_ – 50 K.^37^ VTF fit parameters are shown in Tables 1 and S10. For all PEs, the variation of σ with temperature followed VTF behavior, indicating that ion transport is assisted by the dynamics of the polymer matrix, as typical of PEs above their *T*_g_. To account for the different *T*_g_ values of the PEs, σ values were plotted as a function of (*T*–*T*_g_)^−1^ (Figure 3b). In this plot, all points overlap for different PEs if the differences in their ionic conductivities are governed by variations in segmental motion, which are sufficiently described by the *T*_g_.^21,38^ Thus, the plot in Figure 3b highlights that the average segmental dynamics expressed by *T*_g_ cannot fully explain the different ionic conductivities of P­(OEG)­MAs with varied side-chain distributions.

Bennington et al. have demonstrated that trends in ionic conductivity for similar P­(OEG)_p_MAs synthesized from (OEG)_p_MA-300 and (OEG)_p_MA-500 are primarily governed by side-chain length, which outweighs the effect of *T*_g_.^21^ As the EO units located far from the backbone display increased segmental dynamics, they are more effective in promoting ion transport, leading to higher σ values for P­(OEG)_p_MA with longer side chains. Of note, ∼25 mol % of the species in (OEG)_p_MA-500 have side chains with the same or lower number of EO units than those found in (OEG)_p_MA-300 (4–6 units, Table S1) and therefore do not effectively contribute to the conductivity enhancement. MD simulations of graft polymethacrylates with 9 side-chain EO units have revealed that units 4–9 (with 9 being the farthest from the backbone) are the most frequently involved in Li^+^ solvation. However, the side-chain heterogeneity of common P­(OEG)_p_MAs was not considered in simulations, thus neglecting the influence of EO units located even further from the backbone.

In the P­(OEG)_p_MA used in this work, nearly half (∼46%, Table S1) of the repeating units have >8 EO segments. Thus, in comparison to P­(OEG)_8_MA, P­(OEG)_p_MA possesses a greater proportion of EO units that effectively participate in ion solvation (Figure 3c). At the same time, a smaller fraction (∼40%) of the repeating units in P­(OEG)_mp_MA have >8 EO segments, yet approximately one-sixth of those contain more EO units than the longest side chains of P­(OEG)_p_MA (i.e., >15 EO units). The presence of such long side chains (Figure 3c) likely plays a major role in enhancing the conductivity of P­(OEG)_mp_MA.

Additionally, the longer side chains in P­(OEG)_mp_MA are rather diluted, which may further enhance their mobility and involvement in the solvation site formation. Ji et al. reported that OEG side-chain dilution by copolymerization of (OEG)_p_MAs with methyl methacrylate (MMA) generally determines a decrease in ionic conductivity, due to disruption of solvation site connectivity.^22^ However, MMA has a poor Li-ion coordination ability and slow dynamics, thus negatively impacting σ. Conversely, the manipulation of the OEG side-chain dispersity allows for diluting longer side chains comprising a greater proportion of highly mobile EO units with shorter side chains still capable of promoting Li-ion transport, albeit to a lower extent. Furthermore, broadening side-chain distribution while keeping a constant *n*_av,EO_ of approximately 8 limits the increase in crystallinity caused by the use of P­(OEG)_p_MAs with longer side chains.^22^

To support the hypothesis that the presence of long chains is critical for enhancing the ionic conductivity of these graft PEs, we prepared a polymer using a macromonomer mixture composed primarily of (OEG)_p_MA-300 (75 mol %), with the addition of 23 mol % (OEG)_p_MA-950 and 2 mol % (OEG)_p_MA-500 to maintain *n*_av,EO_ = 8.5. The ionic conductivity of the corresponding PE was an order of magnitude higher than that of an analogous PE composed exclusively of (OEG)_p_MA-300 ^21,22^ and was comparable to that of P­(OEG)_p_MA (i.e., composed exclusively of (OEG)_p_MA-500, Figure S25).

Aiming to understand whether the enhanced ion transport promoted by side-chain heterogeneity is influenced by the flexibility of the polymer backbone, we synthesized polyacrylates with tailored side-chain-length distributions. Analogously to (OEG)_p_MA, (oligo ethylene glycol) methyl ether acrylate with *M*_*n*_ ∼ 480 g mol^–1^, (OEG)_p_A-480, comprises species with varying number of EO units in the side chains. UPLC and HPLC-ESI/MS revealed a similar side-chain distribution to that of (OEG)_p_MA-500, with EO units ranging from 3 to 15, *M*_*n*,calc_ = 461 ± 117 g mol^–1^, and the highest abundance (16 mol %) of the macromonomer with 8 EO lateral segments, (OEG)_8_A (Tables S6 and S7 and Figure S15). Upon separation of (OEG)_8_A by flash chromatography, the polyacrylates with discrete and disperse OEG side chains, P­(OEG)_8_A and P­(OEG)_p_A, were synthesized by ARGET ATRP. The two polymers featured similar *M*_*n*_ of ∼33 kDa, *D̵* < 1.2 (Table 1 and Figure S22), and *n*_av,EO_ = 8.3 and 8.8 for P­(OEG)_8_A and P­(OEG)_p_A, respectively (Figures S20 and S21).

The temperature-dependent ionic conductivity of the polyacrylate-based PEs (*r* = 0.08) is shown in Figure 4. In contrast to P­(OEG)­MAs, P­(OEG)­A-based electrolytes exhibited no appreciable effect of side-chain dispersity on σ values. At the same time, at *T* = 50 °C, σ more than doubled compared to that of P­(OEG)_p_MA, in agreement with the measurements of Bennington et al.^21^ This difference in conductivity is not captured by the polymers’ *T*_g_ (Tables 1 and S8) but rather originates from the higher flexibility of polyacrylates’ backbone in comparison with polymethacrylates. The faster relaxation of polyacrylates’ backbone leads to more rapid relaxation of the EO units in its proximity than in the corresponding polymethacrylates.^21^ Thus, we primarily attribute the negligible influence of side-chain dispersity to accelerated backbone relaxation, which minimizes the position dependence of EO segmental dynamics.

**Figure 4:**
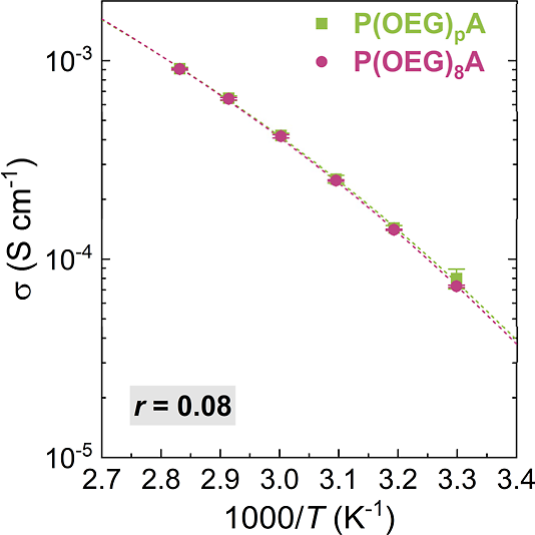
Ionic conductivity (σ) values measured by EIS of PEs comprising LiTFSI (*r* = [Li^+^]/[EO] = 0.08) and polyacrylates with discrete and disperse OEG side chains. σ values were fitted using the VTF equation, where the Vogel temperature *T*_0_ = *T*_g_ – 50 K.

To gain further insight into the relations between side-chain dispersity, polymer mobility, and ion transport, the viscoelastic response of P­(OEG)­MAs and the corresponding PEs was studied by oscillatory rheometry in the linear regime (strain 1%) at 25 °C. Neat P­(OEG)_8_MA and P­(OEG)_p_MA exhibited “liquid-like” viscoelastic behavior, with the gap between *G*″ and *G*′ decreasing at higher frequency (Figure S26). For neat P­(OEG)_mp_MA, *G*′ showed a weak dependence on frequency and it was higher than *G*″ at relatively low frequencies, indicating an elastic behavior, which is attributed to the interpenetration of longer chains. Upon addition of salt (*r* = 0.08) to P­(OEG)_mp_MA, *G*′ shows a more pronounced frequency dependence and greater values at relatively high frequencies, as salt interactions enhance the elasticity of polymer chains.^39^ In the explored frequency range, *G*″ > *G*′ denotes a liquid-like behavior. For all PEs, an increase in *G*″ is observed upon addition of salt, as polymer–salt interactions increase the viscosity of the materials. P­(OEG)_mp_MA showed the highest values of *G*″ in the analyzed frequency range.

Structural differences and dynamics of P­(OEG)­MAs were further probed by ^1^H high-resolution nuclear magnetic spectroscopy (HR NMR), in the temperature range of 25–84 °C. For all P­(OEG)­MAs blended with LiTFSI (*r* = 0.08), broad resonances were recorded at 25 °C, which narrowed significantly with increasing the temperature as a consequence of enhanced chain mobility (Figure S27). To obtain sharper lines, ^1^H HR-magic angle spinning (HR-MAS) spectra were acquired for both neat polymers and their blend with LiTFSI. The spectra showed well-resolved peaks even at 25 °C for all neat P­(OEG)­MAs (Figure S28). The addition of salt caused a slight downfield shift of the peak associated with the methylene protons in EO units.^40^ Moreover, a new peak appeared for all polymers at 4.01–4.15 ppm, which is likely associated with the –C**H**_**2**_ close to the ester group in EO unit 1. Interestingly, at 65 °C, P­(OEG)_8_MA showed sharp peaks, which were broader for P­(OEG)_p_MA and for P­(OEG)_mp_MA (Figure S29). This trend indicates that P­(OEG)_8_MA chains remain highly mobile upon introduction of Li salt, whereas mobility decreases for the polymers with heterogeneous side chains.

The longitudinal (spin–lattice, *T*_1_) relaxation time for protons, providing information about their rotational mobility, are reported in Table S11. The *T*_1_ lowest values are obtained for P­(OEG)_mp_MA and its corresponding PE. We hypothesize that this relatively fast short-range rotational motion originates from the high proportion of short (4–6 EO units) side chains in P­(OEG)_mp_MA. Pulsed-gradient spin–echo (PGSE) NMR experiments were conducted to measure the diffusion coefficients (*D*s) of the neat polymers and their blends with LiTFSI. P­(OEG)_8_MA exhibited the higher value of *D* in comparison with the other polymers (Table S12), suggesting that the interdigitation of relatively long chains in P­(OEG)_p_MA and P­(OEG)_mp_MA hinders the translational motion of the polymers. The addition of Li-salt resulted in nearly unchanged or lowered *D* values, due to Li^+^ coordination that slows down segmental motion.

Rheological and diffusivity studies thus indicated that P­(OEG)­MAs with enhanced side-chain heterogeneity show higher viscosity and polymer–polymer and polymer–ion interactions than P­(OEG)_8_MA. This is reflected in the lower *T*_g_ of P­(OEG)_8_MA blended with LiTFSI. The opposite trend measured for ionic conductivity, with σ values increasing with side-chain heterogeneity, indicates that overall polymer dynamics do not effectively account for ion transport in graft PEs, in agreement with the findings of Bennington et al.^21^

PEs with different contents of Li salt (*r* = 0.02, 0.05, and 0.1) were also prepared and their conductivity was measured at *T* ranging from 30 to 80 °C (Figures 5a and S30 and Table S13). All PEs exhibited the typical trend for PEO-based electrolytes, whereby σ increases with increasing *r* up to a threshold value beyond which the high content of Li^+^ forming cross-links among chains strongly diminishes their mobility and reduces free volume, hindering intrachain and interchain hopping.^41^ For P­(OEG)_8_MA and P­(OEG)_p_MA, σ at *r* = 0.08 is lower than that at *r* = 0.05, particularly at relatively low temperatures. In contrast, P­(OEG)_mp_MA with *r* = 0.05 and 0.08 showed similar σ values at all temperatures, and the effect of excessive cross-linking became evident only at *r* = 0.1 (Figure 5). Moreover, P­(OEG)_mp_MA exhibited σ values higher than those of the PEs with less heterogeneous side chains at all *r* values (Figure 5b). In contrast, P­(OEG)_8_A and P­(OEG)_p_A exhibited very similar σ values at all salt contents and temperatures (Figure S30).

**Figure 5:**
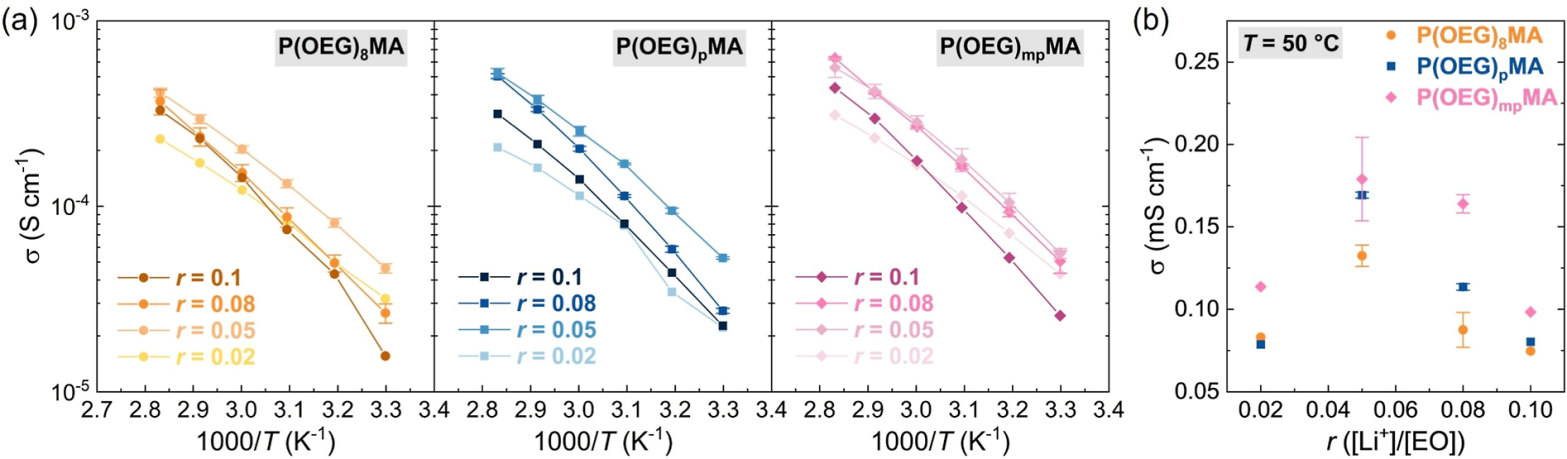
(a) Variation of ionic conductivity (measured by EIS) with the amount of LiTFSI (expressed as *r* = [Li^+^]/[EO]) for PEs based on polymethacrylates with discrete and increasingly more disperse OEG side chains. (b) Comparison of σ values as a function of *r* for the different PEs at *T* = 50 °C.

At *r* = 0.05, P­(OEG)­MAs showed higher ionic conductivity than reported literature values for PEO at *T* < 50 °C.^21,30^ In particular, P­(OEG)_p_MA and P­(OEG)_mp_MA exhibited approximately 2- and 3-fold higher σ values, respectively, compared to PEO (with *M*_*n*_ above its entanglement molecular weight) at *T* = 30 °C, underscoring the advantages of employing graft PEs for room-temperature applications.

To further elucidate the influence of the OEG side-chain dispersity on ion transport, we used ^7^Li and ^19^F pulsed gradient spin echo (PGSE) NMR to measure the self-diffusion coefficients of Li^+^ and TFSI^–^ ions, respectively, in the temperature range of 30–80 °C for P­(OEG)_p_MA and P­(OEG)_mp_MA with *r* = 0.08 (Figures 6 and S31). While *D*_anion_ was very similar for both PEs, *D*_cation_ was slightly higher for P­(OEG)_p_MA. This is consistent with rheological data showing enhanced polymer–ion interactions for the polymer with more heterogeneous side chains. The negligible influence of polymer structure on *D*_anion_ indicates that polymer chains are predominantly interacting with Li ions.^42^

**Figure 6:**
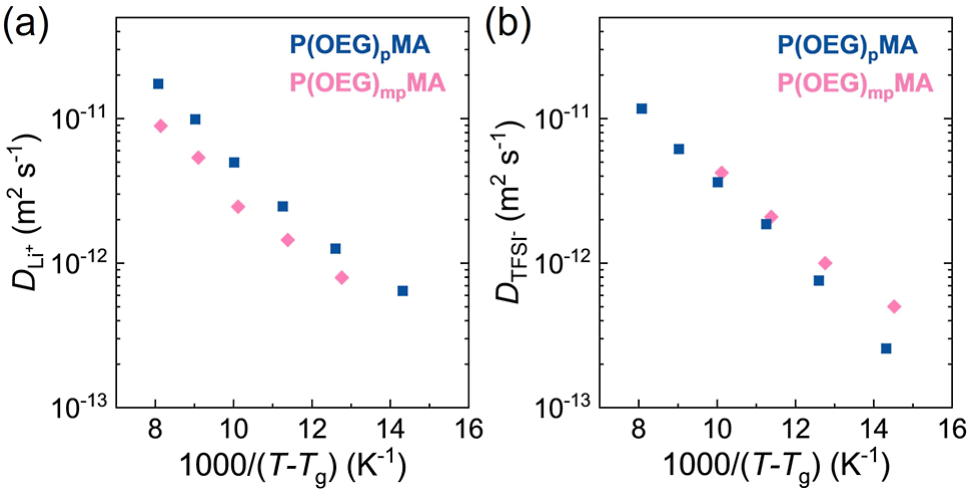
Diffusion coefficients of (a) Li^+^ (^7^Li) and (b) TFSI^–^ (^19^F) as a function of reduced temperature (*T*–*T*_g_), measured by PGSE NMR across the temperature range 25–84 °C for P­(OEG)_p_MA- and P­(OEG)_mp_MA-based electrolytes with *r* = 0.08.

The activation energy for cation and anion diffusion (*E*_a,diff_) was determined by fitting the temperature dependence of the diffusion coefficients to the Arrhenius equation:

2 $$D\left(T\right)=D_{0}\exp\left(-\frac{E_{a}}{RT}\right)$$

where *D*_0_ is a pre-exponential factor (Figure S31). The *E*_a,diff_ values for the diffusion of TFSI^–^ were 62.2 ± 4.5 and 60.0 ± 1.1 kJ mol^–1^ for P­(OEG)_p_MA and P­(OEG)_mp_MA, respectively, whereas for Li^+^ diffusion, *E*_a,diff_ = 55.8 ± 1.1 and 51.2 ± 2.2 kJ mol^–1^ for P­(OEG)_p_MA and P­(OEG)_mp_MA, respectively. For both PEs, the activation energy associated with TFSI^–^ diffusion is higher than that of Li^+^, which can be attributed to the distinctive transport mechanism of Li^+^ ions, whose motion is coupled to the segmental dynamics of the polymer matrix.^36^

The self-diffusion coefficients of Li^+^ and TFSI^–^ ions can be used to estimate the inverse Haven ratio, which is typically interpreted as the degree of dissociation (α) of the Li salt.^42–44^ This is obtained by comparing σ measured by EIS to the conductivity calculated by the Nernst–Einstein equation (eq 3), using *D*_cation_ and *D*_anion_ measured by NMR:

3 $$\sigma_{NMR}=\frac{F^{2}}{RT}\sum c_{i}z_{i}D_{i}$$

where *c*_*i*_ is the molar concentration of the ions, *z*_*i*_ is their absolute charge, *D*_*i*_ is their diffusion coefficient, and *F* is the Faraday constant. Thus, the Nernst–Einstein equation provides conductivity values in the ideal case of complete salt dissolution and absence of ion aggregation, i.e., when all species contributing to ion self-diffusion are also responsible for measured conductivity values.^43,45^ The degree of salt dissociation is then defined as α = σ/σ_NMR_. Values of α below unity are common in PEs, and they are attributed to neutral ion pairs that do not contribute to conductivity under an applied electric field, but their motion contributes to the diffusion coefficients measured by NMR.^43^ Relevantly, P­(OEG)_mp_MA showed α = 0.84–1, whereas P­(OEG)_p_MA exhibited a lower value of α = 0.50–0.64 (Table S14). Thus, LiTFSI is substantially more dissociated in the polymer with more heterogeneous OEG side chains. As the salt content is increased in PEs, ion aggregation also tends to increase.^46,47^ However, LiTFSI remained largely dissociated for P­(OEG)_mp_MA at *r* = 0.08, contributing to maintaining relatively high conductivity values.

## 3 Conclusion

The role of structural dispersity in graft PEs bearing EO units in their side chains was investigated. PEs with uniform side chains (*D̵* = 1.00) were synthesized by isolating discrete (meth)­acrylate macromonomers with 8 EO units in the lateral segments. To broaden the distribution of side-chain length in P­(OEG)­MAs, macromonomers with different compositions were blended to obtain a side-chain dispersity of *D̵* > 1.2.

Ionic conductivity increased with an increase in the heterogeneity of the OEG side chains in graft polymethacrylate electrolytes. This trend was not paralleled by similar variations in *T*_g_ or polymer diffusivity, indicating that overall polymer dynamics have a limited influence on ion transport. The presence of relatively long side chains in P­(OEG)­MAs with higher heterogeneity provides a large fraction of EO units that effectively participate in ion solvation, determining higher σ values. Additionally, long side chains are diluted along the backbone by shorter, yet ion-coordinating, side chains, which limits their tendency to crystallize without penalizing conductivity through the introduction of noncoordinating spacer moieties. These effects, however, become negligible in analogous polyacrylate electrolytes, in which the local dynamics of EO units are overridden by the enhanced flexibility of the backbone.

For more rigid polymethacrylate electrolytes, the polymer with the highest side-chain heterogeneity, P­(OEG)_mp_MA, enabled greater dissociation of Li salt and thus maintained high σ values when increasing the salt content from *r* = 0.05 to *r* = 0.08, whereas other PEs showed a marked decrease in conductivity.

The dominant effect of local EO-unit dynamics, rather than overall polymer dynamics, on ion transport in P­(OEG)­MAs makes side-chain heterogeneity a key structural feature. These findings thus show that structural dispersity of graft polymers can be used as a tool to modulate ion transport and ultimately design PEs with improved performance.

## 4 Methods

### 4.1 Isolation of Discrete (OEG)_8_MA and (OEG)_8_A

(OEG)_p_MA was dissolved in the eluent (98/2 EtOAc/MeOH), and (OEG)_8_MA was isolated by flash chromatography using silica gel under nitrogen pressure. Single-fraction detection was performed using SiO_2_-coated TLC sheets stained with a KMnO_4_ solution and the previously mentioned mobile phase. Solutions of discrete macromonomer fractions were dried in a rotavapor under reduced pressure after the addition of hydroquinone, which acts as an inhibitor. Both commercial macromonomers and (OEG)_8_MA were characterized by ^1^H NMR and UPLC and stored at −20 °C. The same procedure was used to isolate (OEG)_8_A from (OEG)_p_A.

### 4.2 Polymerization of (OEG)­MAs and (OEG)­As

P­(OEG)_8_MA, P­(OEG)_p_MA, P­(OEG)_mp_MA, and P­(OEG)_X_MA were synthesized by activators regenerated by electron transfer atom transfer radical polymerization (ARGET-ATRP). In a representative polymerization, CuBr_2_ (22.3 mg, 0.10 mmol) and TPMA (32.3 mg, 0.11 mmol) were dissolved in 2 mL of DMF. Then, (OEG)_8_MA (1.07 g, 2.37 mmol), Milli-Q H_2_O (3.96 mL), HEBiB (5 μL, 0.03 mmol), NaBr (23.1 mg, 0.23 mmol), and 0.13 mL of the solution of CuBr_2_/tris­(2-pyridylmethyl)­amine (TPMA) were added into a Schleck flask. The solution was degassed with Ar for 30 min. Separately, l-ascorbic acid (AscAc, 26.4 mg, 0.15 mmol) was dissolved in 5 mL of Milli-Q H_2_O in a round-bottom flask, and the solution was degassed with Ar for 30 min. To start the polymerization, 35 mL (0.15 equiv relative to CuBr_2_) of the solution of AscAc was withdrawn with a degassed syringe and injected into the polymerization solution. During polymerization, five aliquots of the solution of AscAc were withdrawn with a degassed syringe and injected into the polymerization solution at regular 1 h-intervals. The monomer conversion and evolution of polymer molar mass were monitored by withdrawing samples during the polymerization and analyzing them with ^1^H NMR and GPC, respectively. When the desired conversion was reached, the polymerization was stopped by opening the vials to the air. Then, the polymerization solution was mixed with THF and passed through a column filled with neutral alumina to remove the Cu catalyst. The solution was then dialyzed (MWCO 1 kDa) against water for 2 days. The final polymer was freeze-dried to remove water, dried in an oven at 80 °C, and kept in a fridge. The effective removal of solvents and monomer was verified by ^1^H NMR. The polymerization of (OEG)As followed a similar procedure, except that NaBr was not introduced into the polymerization mixture and AscAc was introduced all at once at the beginning of the polymerization (0.5 equiv relative to Cu).

### 4.3 Preparation of Polymer Electrolytes

Polymer electrolytes were prepared by mixing the purified polymer P­(OEG)_8_MA, P­(OEG)_p_MA, P­(OEG)_mp_MA, P­(OEG)_X_MA, P­(OEG)_8_A, and P­(OEG)_p_A with LiTFSI. When small amounts of polymers were used, the salt was dissolved in anhydrous THF and the required amount of solution was withdrawn and added to the polymer. The mixture of polymers and salt were dried overnight in an oven at 80 °C. Polymer electrolyte samples were stored in an Ar-filled glovebox (relative humidity <0.1% and O_2_ content <20 ppm). The amount of salt was calculated according to the desired *r* value, expressed as *r* = [Li^+^]/[EO]. The following molar masses of the macromonomers and (average) number of EO units were used for the different polymers: (i) P­(OEG)_8_MA: *M*_*n*_ = 452 g mol^–1^, #EO = 8; (ii) P­(OEG)_p_MA: *M*_*n*_ = 466 g mol^–1^, #EO = 8.4; (iii) P­(OEG)_mp_MA: *M*_*n*_ = 471 g mol^–1^, #EO = 8.5; (iv) P­(OEG)_X_MA: *M*_*n*_ = 471 g mol^–1^, #EO = 8.5. As a result, the fraction of salt in each polymer electrolyte was approximately 9.2–9.3, 20.3–20.6, 28.9–29.3, and 33.7–34.1 wt % for *r* = 0.02, 0.05, 0.08, and 0.1, respectively. For polyacrylates: (i) P­(OEG)_8_MA: *M*_*n*_ = 438 g mol^–1^, #EO = 8; (ii) P­(OEG)_p_A: *M*_*n*_ = 461 g mol^–1^, #EO = 8.5. As a result, the fraction of salt in each polymer electrolyte was approximately 9.5–9.6, 20.8–20.9, 29.6–29.8, and 34.4–34.6 wt % for *r* = 0.02, 0.05, 0.08, and 0.1, respectively.

## Supplementary Material



## Notes

Detailed list of employed materials and instruments, characterizations of macromonomers and polymers via HPLC-ESI/MS, ^1^H NMR, GPC, and TGA, polymerization kinetics, rheology data, and HR and HR-MAS ^1^H NMR data and spectra (PDF)
