# Supplementary material for: Structural Dispersity as a Determinant of Li-Ion Transport in Ethylene-Oxide-Based Graft Polymer Electrolytes
Source: Chem Mater. 2026 Feb 6;38(4):2063–71. doi: 10.1021/acs.chemmater.5c03475 (PMC12937188; doi:10.1021/acs.chemmater.5c03475)
Supplement: Supplementary file 1 [file cm5c03475_si_001.pdf]

Structural Dispersity as a Determinant of Li-Ion Transport in Ethylene-Oxide Based Graft Polymer Electrolytes

Anna Vigolo[1], Valeria Vanoli[2], Luca Laugeni[3], Carlos Pavòn[1], Rossana Pasquino[3], Edmondo M. Benetti[1], Franca Castiglione[2]*, Francesca Lorandi[1]*

[1] *Laboratory for Macromolecular and Organic Chemistry, Department of Chemical Sciences, University of Padova, via Marzolo 1, 35131 Padova, Italy.*

[2] *Department of Chemistry, Materials and Chemical Engineering "G. Natta", Politecnico di Milano, Piazza L. da Vinci 32, 20133 Milano, Italy.*

[3] *DICMaPI, Università degli Studi di Napoli Federico II, P. le Tecchio 80, 80125 Napoli, Italy.*

Table of content

1. **Materials** 2
2. **Instruments** 3
3. **Characterization of commercial (OEG)$_p$MAs, of their blend, and of (OEG)$_8$** 6
4. **ARGET ATRP of methacrylates with different distributions of OEG side chains** 10
5. **Characterization of commercial (OEG)$_p$A and of (OEG)$_8$A** 14
6. **ARGET ATRP of acrylates with different distributions of OEG side chains** 17
7. **Additional characterizations of polymers and polymer electrolytes** 19

1. Materials

For polymerizations: Copper(II) bromide ($CuBr_2$, 99%, Merck), tris(2-pyridylmethyl)amine (TPMA, 98%, Merck), 2-hydroxyethyl 2-bromoisobutyrate (HEBIB, 95%, Merck), L-ascorbic acid (AscAc, 99%, Merck), sodium bromide (NaBr, ≥ 99.5%, Merck), silica gel (Macherey-Nagel 60, 230–400 mesh granulometry (0.063-0.040 mm)), basic and neutral alumina (Al_2O_3, standard grade, Merck), potassium permanganate ($KMnO_4$, 99%, Merck), methanol (MeOH, >99.9%, VWR), acetonitrile (ACN, HPLC grade, Merck), ethanol (EtOH, stndard grade, VWR), ethyl acetate (EtOAc, HPLC grade, Merck), N,N-dimethylformamide (DMF, >99%, VWR), dichloromethane (DCM, anhydrous >99.8%, Merck), trifluoroacetic acid (TFA, >99.7%, Merck) were used as received. Deuterated water (D_2O, 99.96 atom % D) and deuterated chloroform ($CDCl_3$, 99.8 atom % D) were purchased from Eurisotop and used as received.

Oligo(ethylene glycol)methyl ether methacrylate ((OEG)$_p$MA) macromonomers with molar mass of ~300 g mol^{-1}, ~500 g mol^{-1}, and ~950 g mol^{-1} ((OEG)$_p$MA-300, (OEG)$_p$MA-500, and (OEG)$_p$MA-950), and oligo(ethylene glycol)methyl ether acrylate ((OEG)$_p$A) with molar mass of ~480 g mol^{-1} were purified to remove the inhibitors by passing through a column filled with basic alumina.

MilliQ water has been obtained through a Millipore Direct-Q5 or Milli-Q® IQ 7003 purification system. TLC sheets (Macherey-Nagel Polygram®SIL G/UV254, silica thickness 0.2 mm, Duren, Germany) were used as received.

For polymer electrolytes: Lithium bis(trifluoromethanesulfonyl)imide (LiTFSI, 99.95%) was kept in a dessicator and oven-dried prior to use.

2. Instruments

Ultra performance liquid chromatography (UPLC). UPLC analysis of macromonomers has been performed using an Agilent 1290 Infinity LC System (Agilent Technologies, Milan, Italy), equipped with a binary pump, a ZORBAX Eclips XDB-C18 column (2.1 x 50 mm, 1.8 μm), and a photodiode array detector (190-250 nm) set at a wavelength of 220 nm. For $(OEG)_pMA$-300, $(OEG)_pMA$-500, $(OEG)_pA$, $(OEG)_8MA$, $(OEG)_8A$, the method consisted in an eluent gradient from 95/5 H_2O/ACN (both with 0.1% TFA) to 100% ACN, during a 25-min run. For $(OEG)_pMA$-950, the method consisted in an eluent gradient from 75/25 H_2O/ACN (both with 0.1% TFA) to 100% ACN, during a 25-min run.

Electrospray ionization/mass spectrometry-high performance liquid chromatography (ESI/MS-HPLC). Low resolution mass spectra of the macromonomers were obtained with a 1100 Series Agilent Technologies system, equipped with a binary pump (G1312A) and MSD SL trap mass spectrometer (G2445D SL). High resolution mass analysis of macromonomers was performed with a Xevo G2-XS Q-ToF. In all cases the instruments were equipped with an ESI source, either in positive or negative mode. Sample solutions were prepared in ACN to achieve a concentration in the order of 10^{-3}–10^{-4} M.

Nuclear magnetic resonance spectroscopy (NMR). ^{1}H NMR and ^{13}C NMR spectra were recorded using Bruker Advanced III 400 MHz or 500 MHz spectrometers at room temperature and using $CDCl_3$ or D_2O as solvent. Chemical shifts (δ) are given in ppm relative to the signal of the residual non-deuterated solvent.

High resolution (HR) NMR spectra of the polymers were acquired on a Bruker NEO 500 console (11.74 T) equipped with a direct observe BBFO (broadband including fluorine) iProbe and a variable-temperature unit (^{1}H resonance frequency of 500.13 MHz). Diffusion coefficients of cation (^{19}F) and anion (^{7}Li) were measured by pulsed gradient spin echo (PGSE) experiments applying sine-shaped pulsed magnetic field gradients along the *z*-direction up to a maximum strength of G = 48.15 G cm^{-1}. The experiments were performed using the bipolar pulse

longitudinal eddy current delay (BPP-LED) pulse sequence.[1] The pulse gradients were incremented from 2 to 95% of the maximum gradient strength in a linear ramp with 32 steps. For each experiment, the duration of the magnetic field pulse gradients (δ) and the diffusion times (Δ) were optimized to obtain 95% signal attenuation at the last step experiment. δ values were 3.0 ms, while Δ values were 0.8 s long. All experiments were performed in the temperature range of 25-84 °C with 8 scans, a relaxation delay D1 of 10 s and sweep width of 10 ppm.

The ^{1}H HR magic angle spinning (MAS) experiments were performed with a dual ^{1}H/^{13}C high-resolution magic angle spinning (HR-MAS) probe head for semisolid samples. The sample was loaded into 4 mm ZrO_2 rotor (12 μL volume). The spectra were acquired at a spinning rate of 4 kHz, sweep width of 10 ppm and 8 scans, and a temperature range of 25-65 °C. The diffusion data were acquired with $\delta = 3.0$ ms, $\Delta = 0.8$ s with a linear ramp gradient of 32 steps. T_1 spin-lattice relaxation times were measured using the inversion recovery (IR) pulse sequence. All spectra were recorded for various delay time τ, in the range 0.01-20 s with data matrices of 16384 (t_2) × 16 (t_1) complex data points over a spectral width of 10 ppm.

Size exclusion chromatography (SEC). SEC was performed using an Agilent 1260 Infinity gel permeation chromatography equipped with a refractive index (RI) detector and two PL gel Mixed-D columns (300 mm, 5 μm) connected in series. The column compartment and RI detector were heated at 70 ºC and 50 ºC, respectively, and the eluent was DMF containing 10 mM LiBr at a flow rate of 1 mL min^{-1}. Every sample contained a polymer concentration of ~2 mg mL^{-1}, and it was filtering through neutral alumina over a PTFE membrane with a porosity of 0.20 μm. To determine the M_n and $Đ$ of the polymers, a calibration curve was built by using 12 poly(methyl methacrylate) standards (M_p = 2.7-400 kDa).

Differential scanning calorimetry (DSC). Experiments were performed with DSC Q250 and DSC Q29 (TA Instrument) in the range −80 to 150 °C, at a heating rate of 10 °C min^{-1}. Different runs were executed to delete previous thermal histories and to check for reproducibility.

Thermogravimetric analysis (TGA). TGA was carried out on a Trios V5.7.1.74 instrument (TA Instrument) in the range 10 to 600 °C, with a heating rate of 10 °C min^{-1}, under nitrogen atmosphere.

Rheometry. Rheological measurements were performed on a stress controlled rotational rheometer MCR 702 (Anton Paar, Graz, Austria) equipped with a Peltier unit for temperature

control. Tests were performed in the linear viscoelastic regime, using the parallel plate geometry with a diameter of 8mm, due to the limited amount of synthesized samples. A gap in the range 1-0.5mm was always used for all experiments. Oscillatory shear tests were performed at 25 °C with an applied strain lower than 10%.

Measurements of Li-ion conductivity. The ionic conductivity of polymer electrolytes was determined on a VIONIC potentiostat/galvanostat with Electrochemical Impedence Spectroscopy (EIS) module (Metrohm), equipped with the INTELLO 1.4 software. An ECC-Std test cell (EL-CELL) was used for the measurements. The cells were assembled inside an argon-filled glovebox to avoid moisture contamination (GS Glovebox Systemtechnik GmbH, relative humidity <0.1% and O_2 content <20 ppm). Symmetrical stainless steel/polymer electrolyte/stainless steel cell was assembled. The distance between the electrodes (l) was kept equal to 100 μm using a Teflon spacer ring with the inner area (A) of 0.503 cm^2. Cell impedance was measured by applying a 10 mV perturbation in the frequency range of 100 kHz to 1 Hz at the open circuit potential (OCV). The ohmic resistance (R_Ω) of the sample, obtained from the Nyquist plot at the low frequency end of the semicircle, was used to calculate the ionic conductivity using the following equation:

$$\sigma = \frac{l}{R_\Omega A}$$

The measurement was carried out between 30 and 80 °C at 10 °C-intervals. The temperature was controlled using an Heratherm convection oven (Thermo Scientific). Cells were allowed to reach the thermal equilibrium for at least 2 h before each test.

3. Characterization of commercial (OEG)pMAs, of their blend, and of (OEG)8

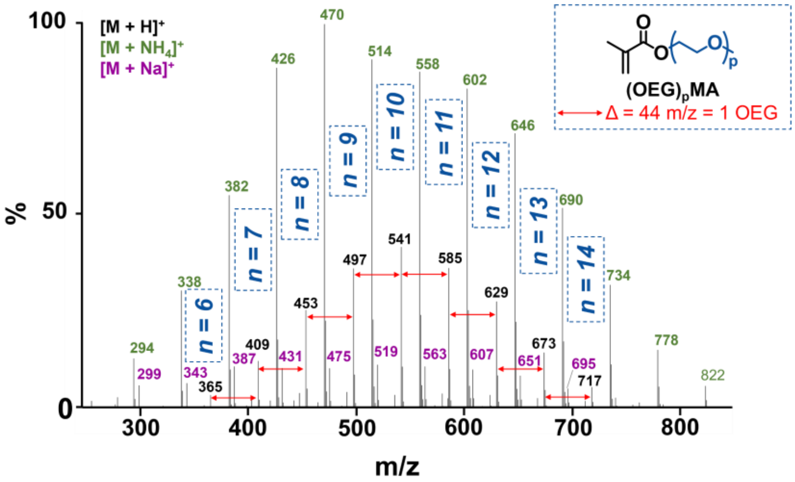

Figure S1. Electrospray ionization/mass spectrometry (ESI/MS) spectrum of commercial $(OEG)_pMA$-500 macromonomer where m/z in black are referred to $[(OEG)_pMA + H]^+$, in green to $[(OEG)_pMA + NH_4]^+$, and in purple to $[(OEG)_pMA + Na]^+$.

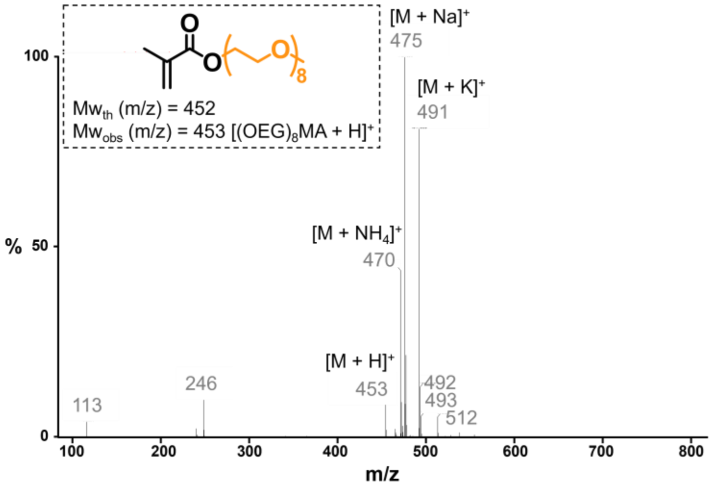

Figure S2. ESI/MS spectrum of the isolated macromonomer by flash chromatography of $(OEG)_pMA$-500. The main product has 8 EO units in the side chains, $(OEG)_8MA$, where m/z = 453 for $[(OEG)_8MA + H]^+$, m/z = 470 for $[(OEG)_8MA + NH_4]^+$, m/z = 475 for $[(OEG)_8MA + Na]^+$, and m/z = 491 for $[(OEG)_8MA + K]^+$.

Table S1. Composition of the different commercial $(OEG)_pMA$ macromonomers and their blend $(OEG)_{mp}MA$ (24 mol% $(OEG)_pMA$-300, 68 mol% $(OEG)_pMA$-500, and 8 mol% $(OEG)_pMA$-950) derived from UPLC and ESI-MS analysis.

# EO units	$(OEG)_pMA$-300	$(OEG)_pMA$-500	$(OEG)_pMA$-950	$(OEG)_{mp}MA$
**2**		0.6 mol%		0.4 mol%
**3**		2.2 mol%		1.5 mol%
**4**	72 mol%	4.6 mol%		20.4 mol%
**5**	23 mol%	7.4 mol%		10.6 mol%
**6**	5 mol%	10.7 mol%		8.5 mol%
**7**		13.7 mol%		9.3 mol%
**8**		14.6 mol%		9.9 mol%
**9**		13.7 mol%		9.3 mol%
**10**		11.4 mol%		7.8 mol%
**11**		8.6 mol%		5.8 mol%
**12**		6.2 mol%		4.2 mol%
**13**		3.4 mol%	1 mol%	2.4 mol%
**14**		1.9 mol%	2 mol%	1.5 mol%
**15**		1 mol%	2 mol%	0.8 mol%
**16**			4 mol%	0.3 mol%
**17**			5 mol%	0.4 mol%
**18**			6 mol%	0.5 mol%
**19**			8 mol%	0.6 mol%
**20**			10 mol%	0.8 mol%
**21**			10 mol%	0.8 mol%
**22**			10 mol%	0.8 mol%
**23**			11 mol%	0.9 mol%
**24**			9 mol%	0.7 mol%
**25**			5 mol%	0.4 mol%
**26**			5 mol%	0.4 mol%
**27**			4 mol%	0.3 mol%
**28**			3 mol%	0.2 mol%
**29**			2 mol%	0.2 mol%
**30**			2 mol%	0.2 mol%
**31**			1 mol%	0.1 mol%

Table S2. Number and weight average molar mass and dispersity of the different commercial $(OEG)_pMA$ macromonomers calculated from their composition in **Figure S1-S2** and **Table S1**.

	$(OEG)_pMA$-300	$(OEG)_pMA$-500	$(OEG)_pMA$-950
M_n (g mol^{-1})	291	466	1056
M_w (g mol^{-1})	293	495	1082
Đ	1.01	1.06	1.03
M_n ± st.dev.[a]	291 ± 25	466 ± 117	1056 ± 169

[a] Standard deviation relative to M_n.

Table S3. Composition of the purified $(OEG)_8MA$ macromonomer derived from UPLC and ESI-MS analysis.

# EO units	$(OEG)_8MA$
7	1.1 mol%
8	98.5 mol%
9	0.4 mol%

Table S4. Characteristics of the macromonomers' blend employed to synthesize $P(OEG)_{mp}MA$, which combined 24 mol% $(OEG)_pMA$-300, 68 mol% $(OEG)_pMA$-500, and 8 mol% $(OEG)_pMA$-950.

	$(OEG)_{mp}MA$
M_n (g mol^{-1})	451.69
M_w (g mol^{-1})	451.76
$Đ$	1.00014
M_n ± st.dev.	452 ± 5

Table S5. Characteristics of the macromonomers' blend employed to synthesize $P(OEG)_{mp}MA$, which combined 24 mol% $(OEG)_pMA$-300, 68 mol% $(OEG)_pMA$-500, and 8 mol% $(OEG)_pMA$-950.

	$(OEG)_{mp}MA$
M_n (g mol^{-1})	471
M_w (g mol^{-1})	571
$Đ$	1.21
M_n ± st.dev.	471 ± 217

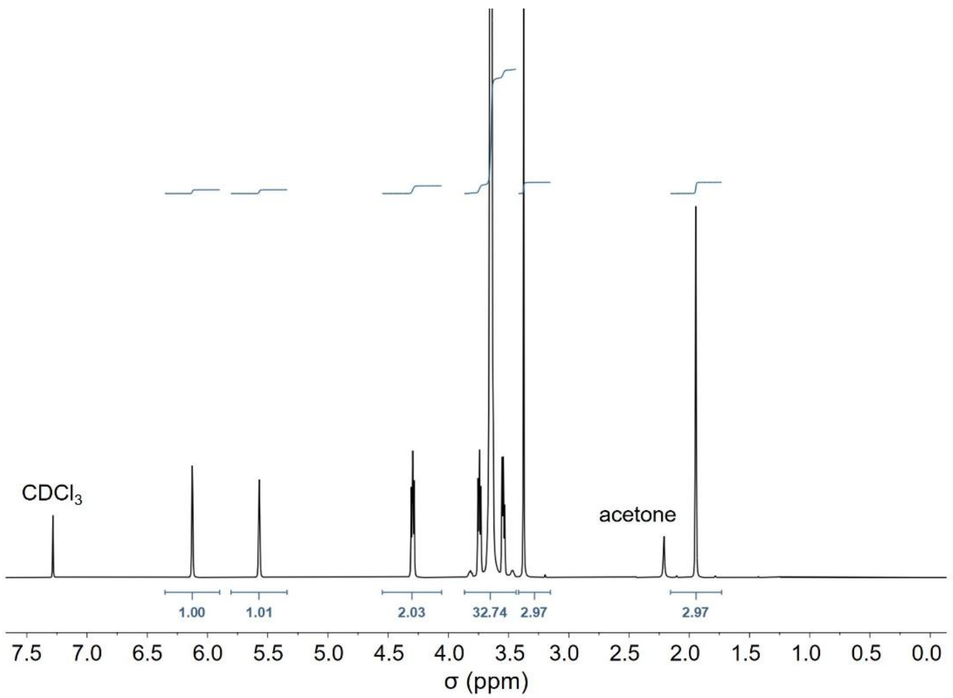

Figure S3. ^{1}H NMR spectrum (400 MHz) of $(OEG)_pMA$-500 macromonomer in $CDCl_3$.

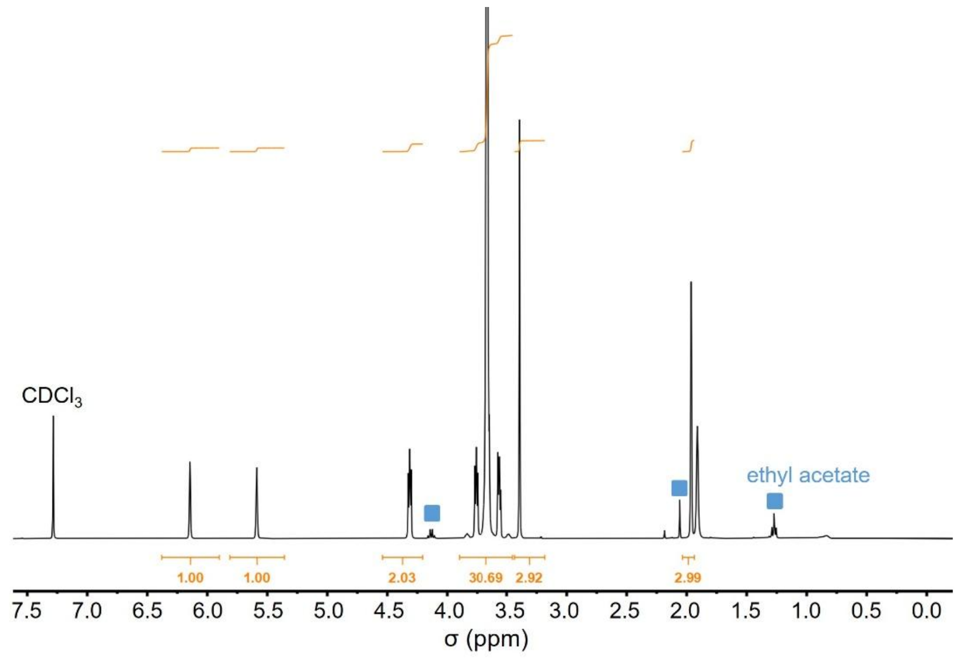

Figure S4. ^{1}H NMR spectrum (400 MHz) of the isolated $(OEG)_8MA$ fraction in $CDCl_3$.

4. ARGET ATRP of methacrylates with different distributions of OEG side chains

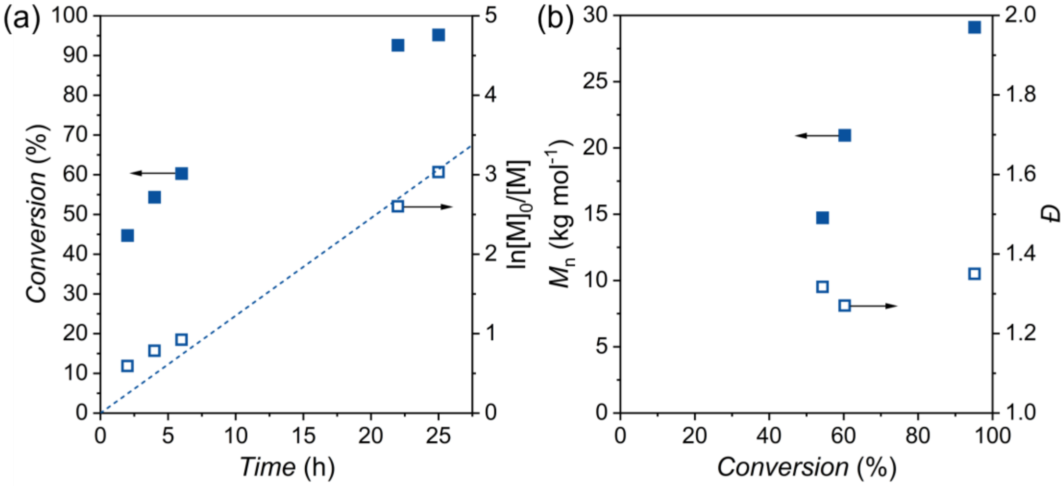

Figure S5. (a) Kinetic plots and (b) evolution of M_n and dispersity with conversion in ARGET ATRP of $(OEG)_pMA$ (20 vol%) in water. Conditions: $[(OEG)_pMA]$:[HEBiB]:$[CuBr_2]$:[TPMA]:[NaBr]:[AscAc] = 70:1:0.2:0.24:10:0.18.

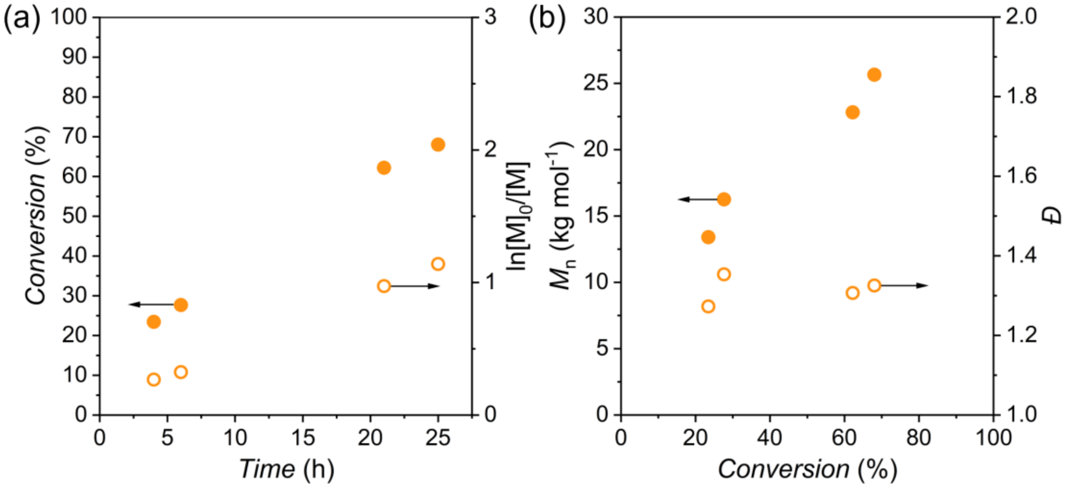

Figure S6. (a) Kinetic plots and (b) evolution of M_n and dispersity with conversion in ARGET ATRP of $(OEG)_8MA$ (20 vol%) in water. Conditions: $[(OEG)_8MA]$:[HEBiB]:$[CuBr_2]$:[TPMA]:[NaBr]:[AscAc] = 100:1:0.15:0.2:7.5:0.09.

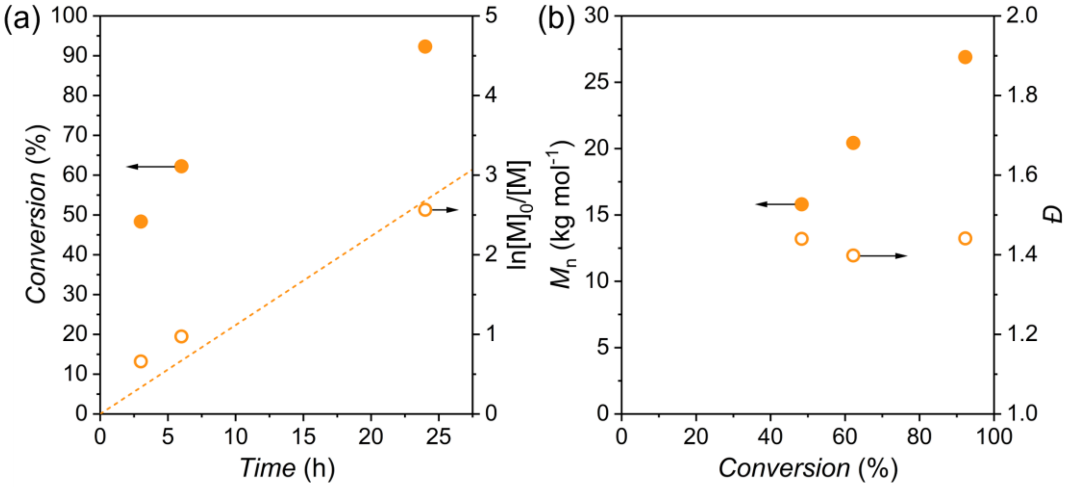

Figure S7. (a) Kinetic plots and (b) evolution of M_n and dispersity with conversion in ARGET ATRP of $(OEG)_8MA$ (20 vol%) in water. Conditions: $[(OEG)_8MA]$:[HEBiB]:$[CuBr_2]$:[TPMA]:[NaBr]:[AscAc] = 70:1:0.2:0.24:10:0.18.

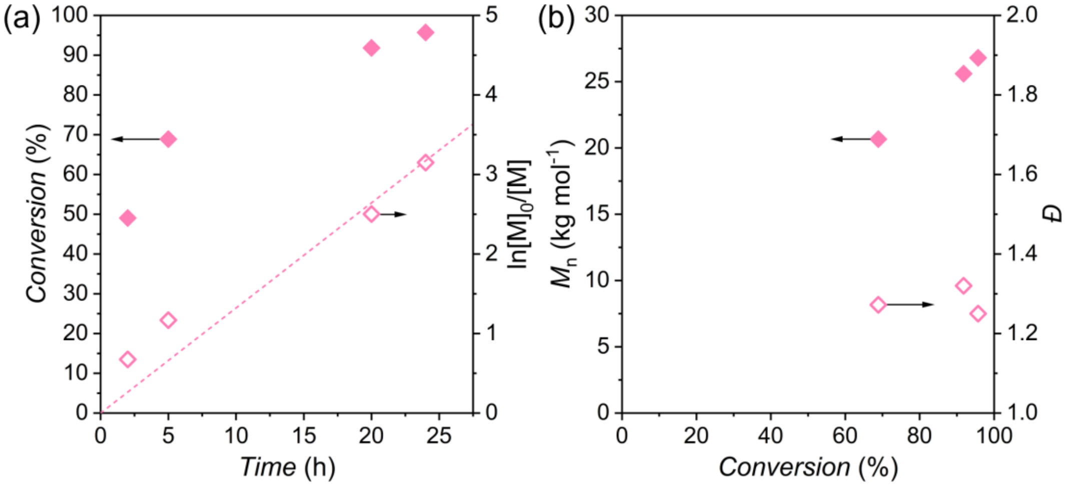

Figure S8. (a) Kinetic plots and (b) evolution of M_n and dispersity with conversion in ARGET ATRP of $(OEG)_pMA$-300 + $(OEG)_pMA$-500 + $(OEG)_pMA$-950 (macromonomer mix = 20 vol%) in water. Conditions: $[(OEG)_pMA$-300]:$[(OEG)_pMA$-500]:$[(OEG)_pMA$-950] = 24:68:8; [macromonomer mix]:[HEBiB]:$[CuBr_2]$:[TPMA]: [NaBr]:[AscAc] = 70:1:0.2:0.24:10:0.18.

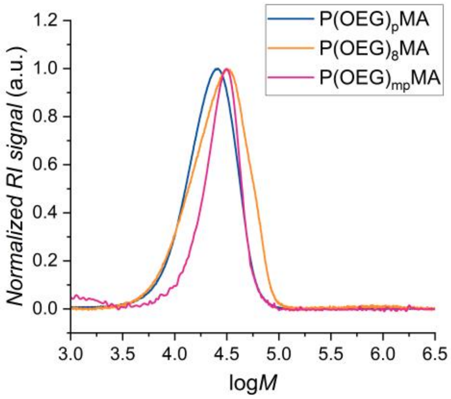

Figure S9. GPC traces of representative polymethacrylates with discrete and disperse OEG side chains synthesized and employed as polymer electrolytes.

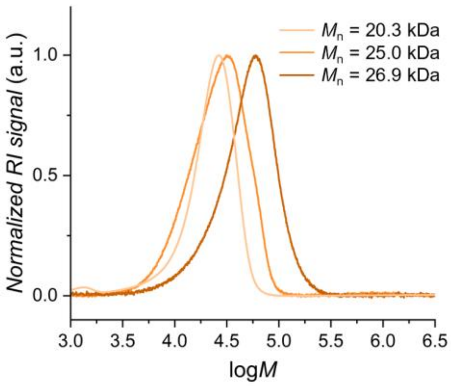

Figure S10. GPC traces of the different P(OEG)$_8$MA synthesized and employed as polymer electrolytes.

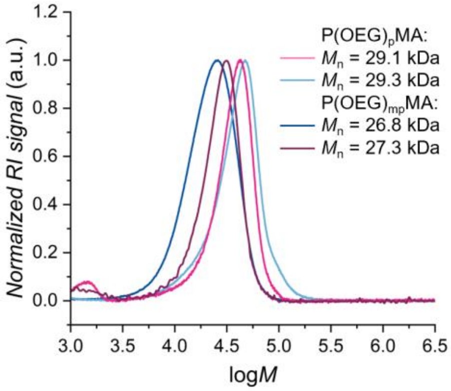

Figure S11. GPC traces of the different P(OEG)$_p$MA and P(OEG)$_{mp}$MA synthesized and employed as polymer electrolytes.

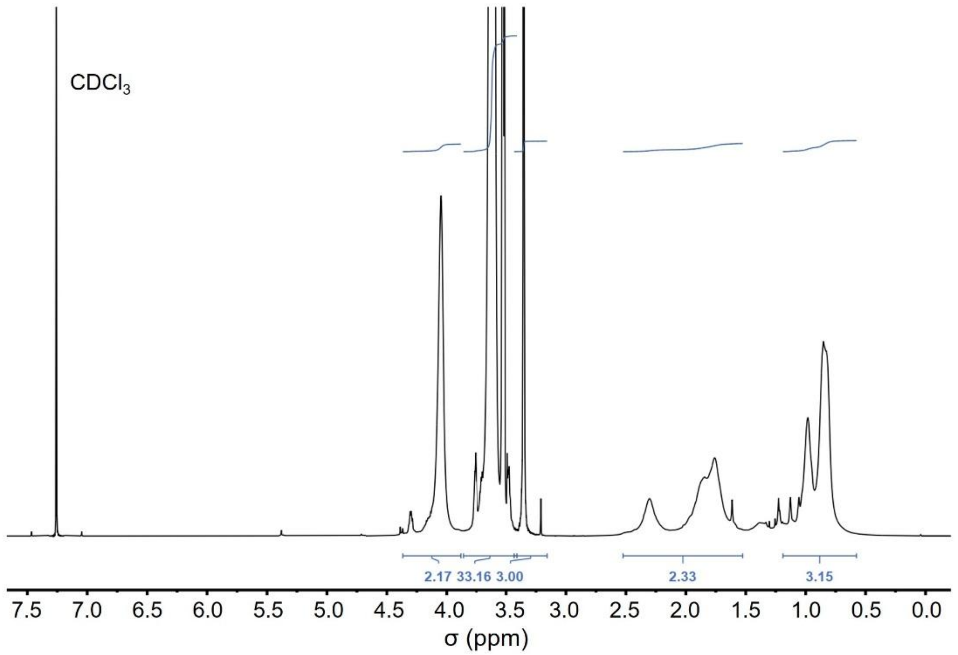

Figure S12. ^{1}H NMR spectrum (500 MHz) of purified $P(OEG)_pMA$ in $CDCl_3$.

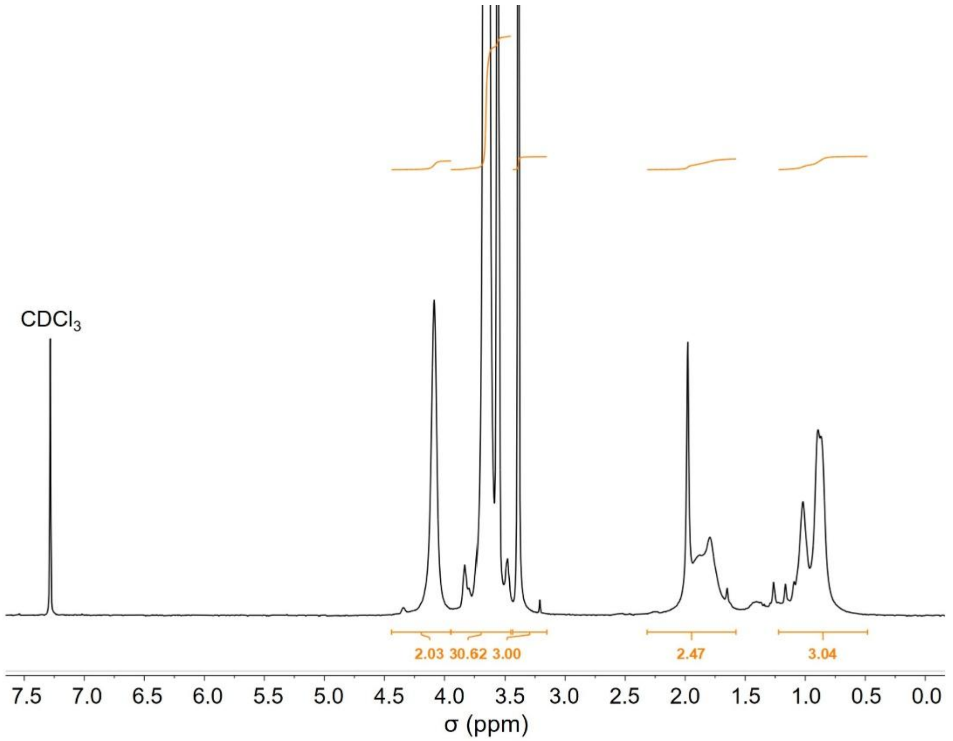

Figure S13. ^{1}H NMR spectrum (400 MHz) of purified $P(OEG)_8MA$ in $CDCl_3$.

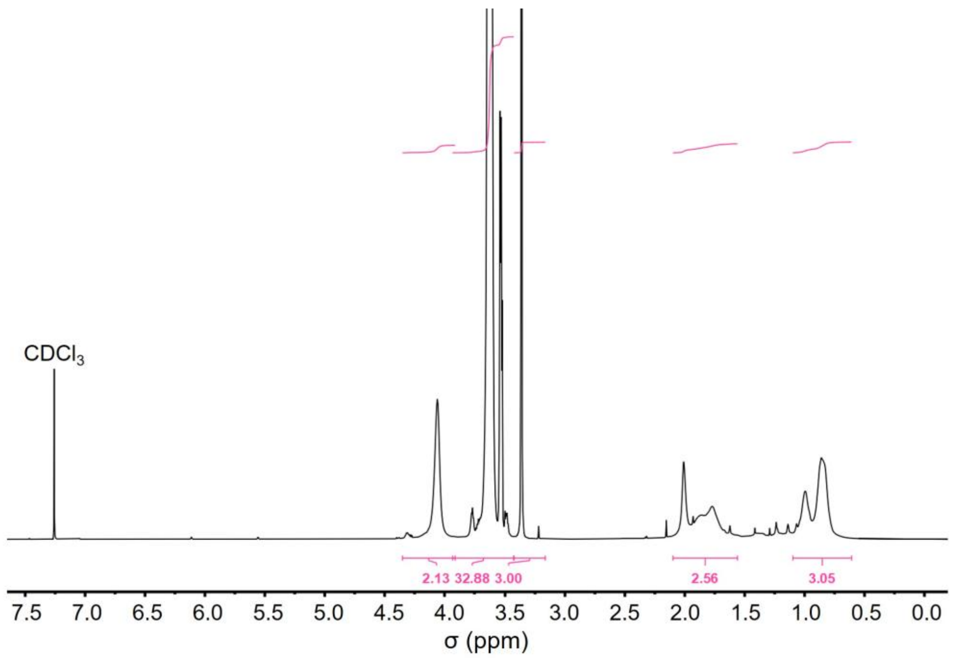

Figure S14. ^{1}H NMR spectrum (500 MHz) of purified $P(OEG)_{mp}MA$ in $CDCl_3$.

5. Characterization of commercial $(OEG)_pA$ and of $(OEG)_8A$

Table S6. Composition of the commercial $(OEG)_pA$ macromonomer (~480 g mol^{-1}) derived from UPLC and ESI-MS analysis.

# EO units	Molar fraction (mol%)
**3**	2
**4**	3
**5**	8
**6**	11
**7**	14
**8**	16
**9**	14
**10**	12
**11**	8
**12**	5
**13**	4
**14**	3
**15**	2

Table S7. Number and weight average molar mass and dispersity of the commercial $(OEG)_pA$ macromonomers calculated from their composition in **Table S6**.

	**$(OEG)_pA$-480**
M_n (g mol^{-1})	461
M_w (g mol^{-1})	490
$Đ$	1.07
M_n ± st.dev. (g mol^{-1})	461 ± 117

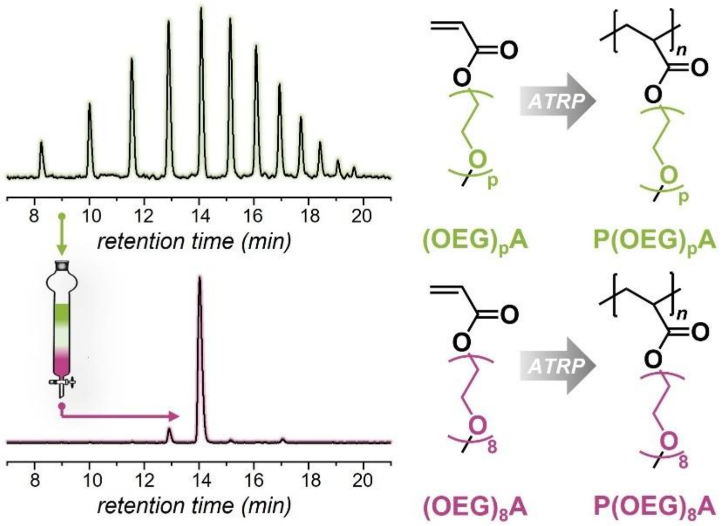

Figure S15. Chemical structure and UPLC elugrams of the commercial macromonomer $(OEG)_pA$ (~480 g mol^{-1}) and the discrete $(OEG)_8A$ fraction separated by flash chromatography, and chemical structure of the corresponding polymers.

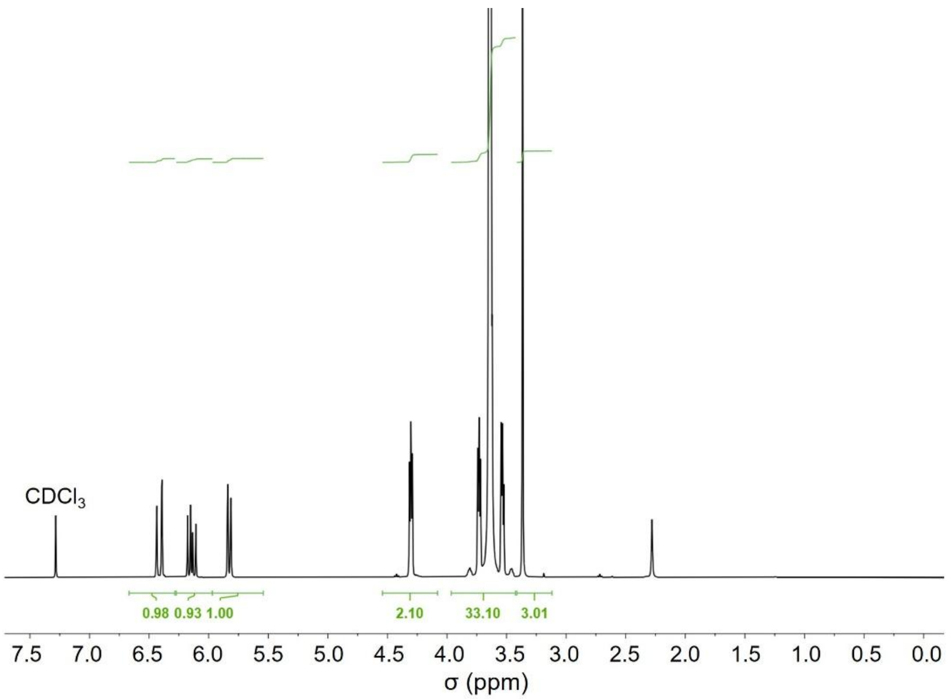

Figure S16. ^{1}H NMR spectrum (400 MHz) of $(OEG)_pA$ (~480 g mol^{-1}) macromonomer in $CDCl_3$.

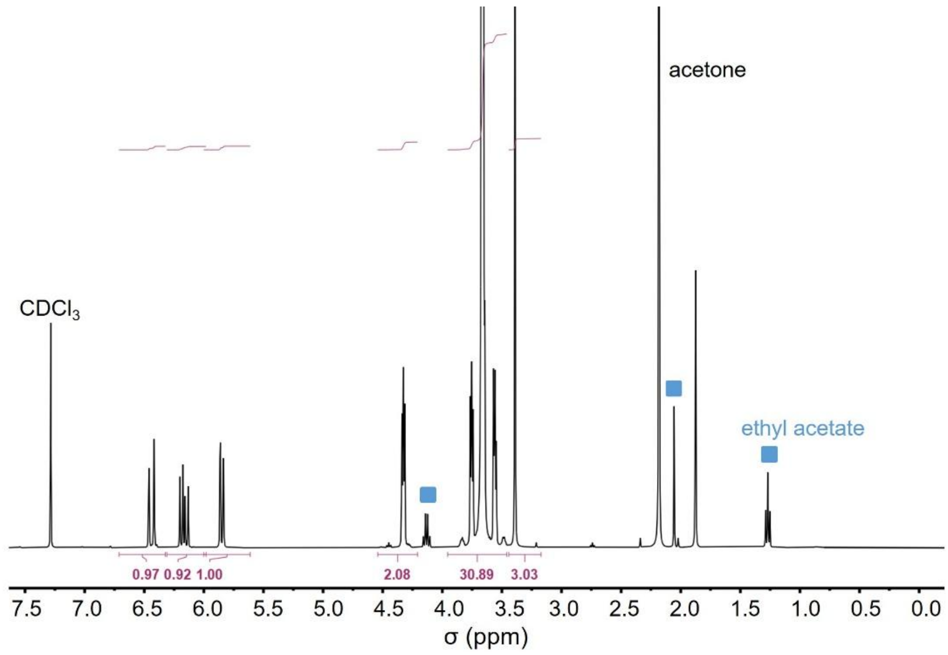

Figure S17. ^{1}H NMR spectrum (400 MHz) of the isolated $(OEG)_8A$ fraction in $CDCl_3$.

6. ARGET ATRP of acrylates with different distributions of OEG side chains

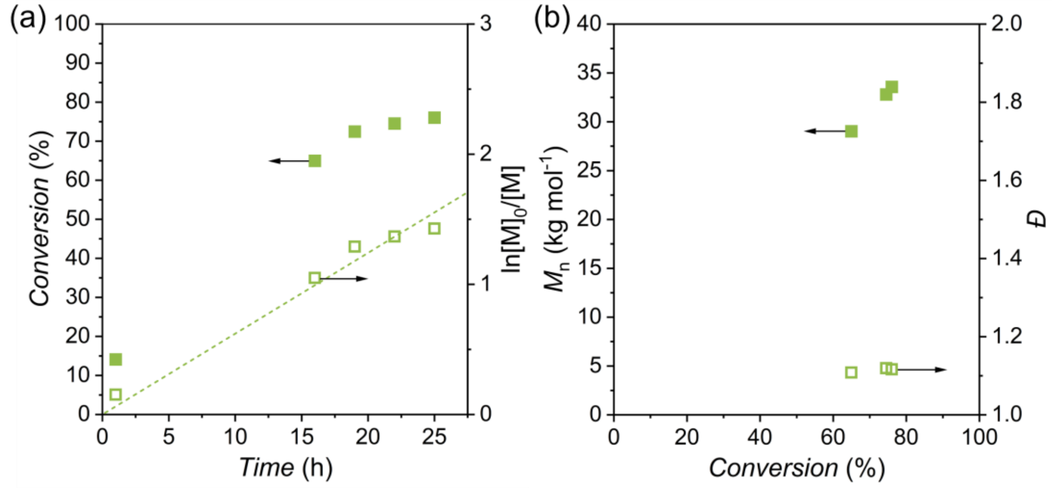

Figure S18. (a) Kinetic plots and (b) evolution of M_n and dispersity with conversion in ARGET ATRP of $(OEG)_pA$ (20 vol%) in water. Conditions: $[(OEG)_pA]:[HEBiB]:[CuBr_2]:[TPMA]:[AscAc]$ = 110:1:0.35:0.42: 0.3.

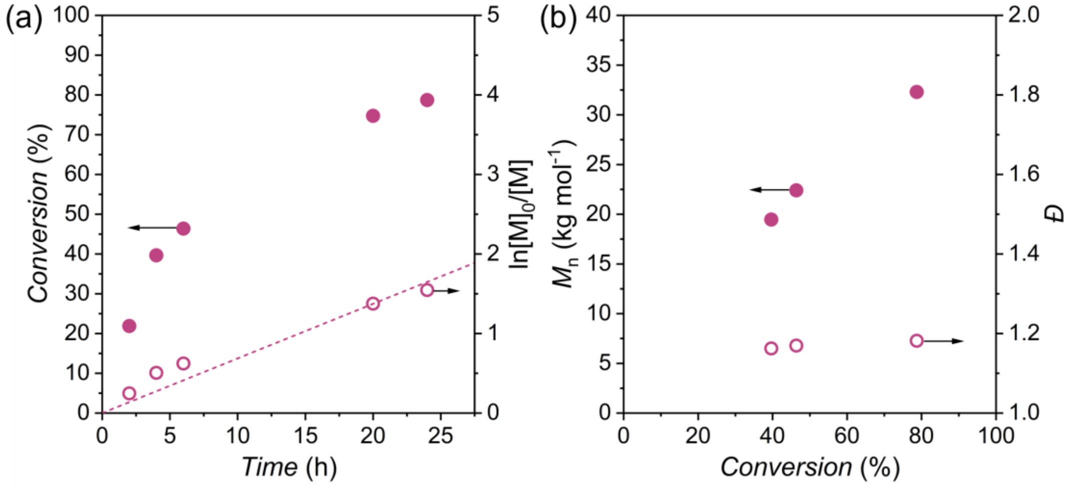

Figure S19. (a) Kinetic plots and (b) evolution of M_n and dispersity with conversion in ARGET ATRP of $(OEG)_8A$ (20 vol%) in water. Conditions: $[(OEG)_8A]:[HEBiB]:[CuBr_2]:[TPMA]:[AscAc]$ = 110:1:0.35:0.42: 0.3.

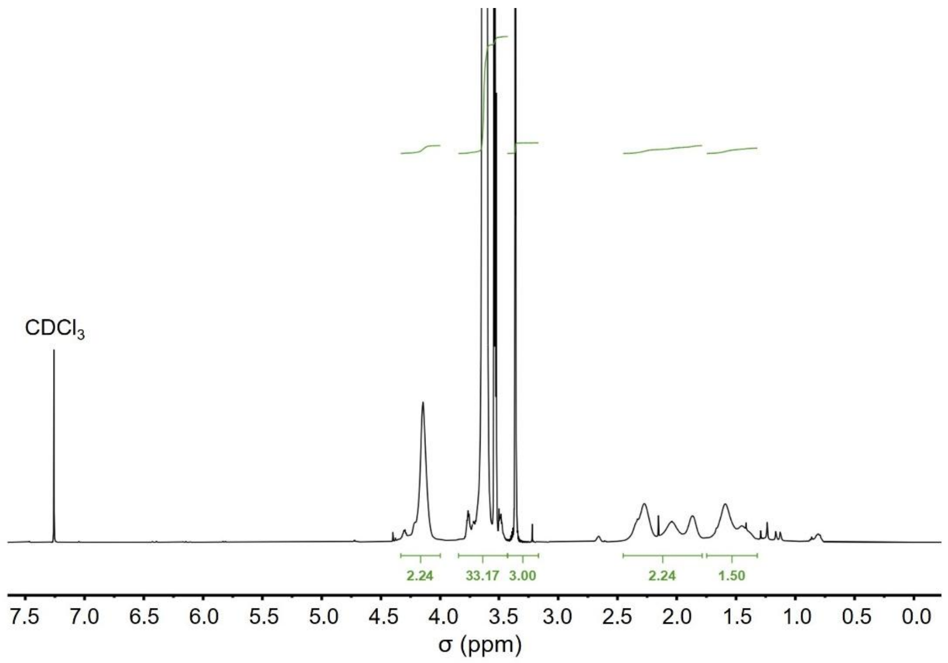

Figure S20. ^{1}H NMR spectrum (500 MHz) of purified P(OEG)$_p$A in $CDCl_3$.

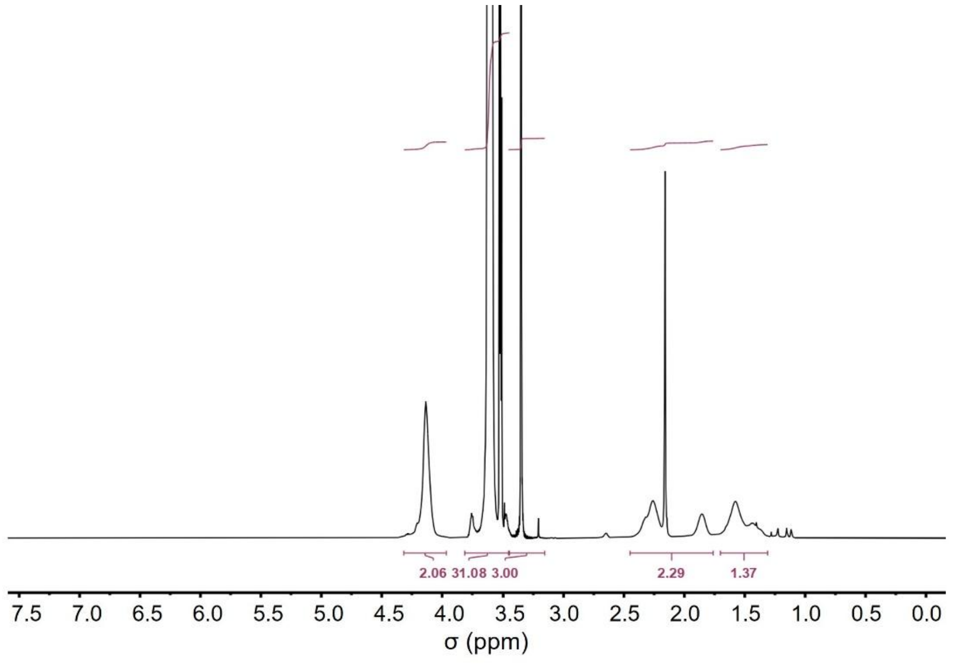

Figure S21. ^{1}H NMR spectrum (500 MHz) of purified P(OEG)$_p$A in $CDCl_3$.

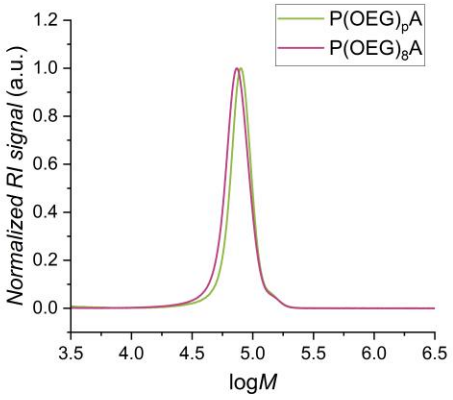

Figure S22. GPC traces of polyacrylates with discrete and disperse OEG side chains synthesized and employed as polymer electrolytes.

7. Additional characterizations of polymers and polymer electrolytes

Table S8. Polymerization and structural data, and T_g in the absence and presence of LiTFSI for all synthesized poly(meth)acrylates with discrete and disperse distribution of OEG side-chain lengths.

**Polymer**	**DP_T**	***t* (h)**	***Conv.* (%)**	**$M_{n,th}$[a] (kg mol^{-1})**	**$M_{n,GPC}$[b] (kg mol^{-1})**	**$M_{n,NMR}$[c] (kg mol^{-1})**	**$Đ$[b]**	**T_g (°C)**	**$T_{g,r=0.08}$ (°C)**
P(OEG)$_8$MA[d]	100	25	68	30.9	25.0	35	1.33	-62	-47
P(OEG)$_8$MA[d]	100	24	62	27.3	26.9	28	1.44	-63	-48
P(OEG)$_8$MA[e]	70	26	86	27.6	20.3	25	1.28	-	-
P(OEG)$_p$MA[d]	100	21	64	30.0	29.3	28	1.31	-60	-41
P(OEG)$_p$MA[e]	70	30	95	31.2	29.1	30	1.35	-61	-40
P(OEG)$_{mp}$MA[e]	70	24	96	31.9	26.8	28	1.25	-63	-
P(OEG)$_{mp}$MA[e]	70	22	99	32.9	27.3	26	1.41	-65	-39
P(OEG)$_x$MA[e]	70	24	95	31.5	29.9	25	1.31	-61	-
P(OEG)$_8$A[gf]	110	24	79	38.2	32.3	32	1.18	-64	-47
P(OEG)$_p$A[gf]	110	25	76	38.7	33.5	34	1.15	-63	-45

[a] Calculted as $M_{n,th} = Conv. \times DP_T \times M_{n,macromonomer(s)} + MM_{HEBiB}$.

[b] Measured by GPC in DMF + 0.01 M LiBr as eluent, using poly(methyl methacrylate) standards.

[c] Estimated by ^{1}H NMR (500 MHz in $CDCl_3$) by integration of the signals of methyl protons in HEBiB.

[d] [Monomer(s)]:[HEBiB]:[$CuBr_2$]:[TPMA]:[NaBr]:[AscAc] = 100:1:0.15:0.2:7.5:0.13.

[e] [Monomer(s)]:[HEBiB]:[$CuBr_2$]:[TPMA]:[NaBr]:[AscAc] = 70:1:0.2:0.22:10:0.18.

[f] [Monomer(s)]:[HEBiB]:[$CuBr_2$]:[TPMA]:[AscAc] = 110:1:0.35:0.4:0.3.

All P(OEG)MAs in **Table S8** were synthesized aiming at a theoretical degree of polymerization of 55-65. This was achieved in two ways: by either targeting a DP of 100 and stopping the polymerization at a conversion of ~60%, or by targeting a DP of 70 and let the polymerization proceed up to relatively high conversion. As it can be appreciated in **Table S8** and in **Figures S5-S8**, the polymerization rate was higher when targeting DP = 70 than when targeting DP = 100, in agreement with the expected behavior of an ATRP.[2]

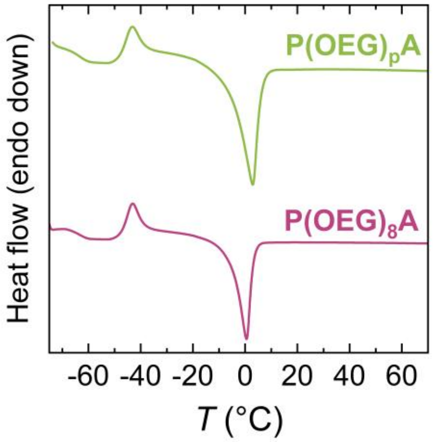

Figure S23. DSC analysis of polyacrylates with discrete and disperse OEG side chains.

Table S9. Thermal properties of representative polymers and the corresponding polymer electrolytes upon mixing with LiTFSI, measured by DSC and TGA.

**Polymer**	T_g (°C)	$T_{g,r=0.08}$ (°C)	T_m (°C)	$T_{m,r=0.08}$ (°C)	$T_{c,c}$ (°C)	T_d (° C)
P(OEG)$_8$MA[d]	-62	-47	-	-		371
P(OEG)$_p$MA[e]	-61	-40	-3.8	-	-21	367
P(OEG)$_{mp}$MA[e]	-65	-39	2.9	-	-30	-
P(OEG)$_x$MA	-61	-	23	-	-40	-
P(OEG)$_8$A[gf]	-64	-47	0.5	-19	-43	386
P(OEG)$_p$A[gf]	-63	-45	2.9	-	-44	386

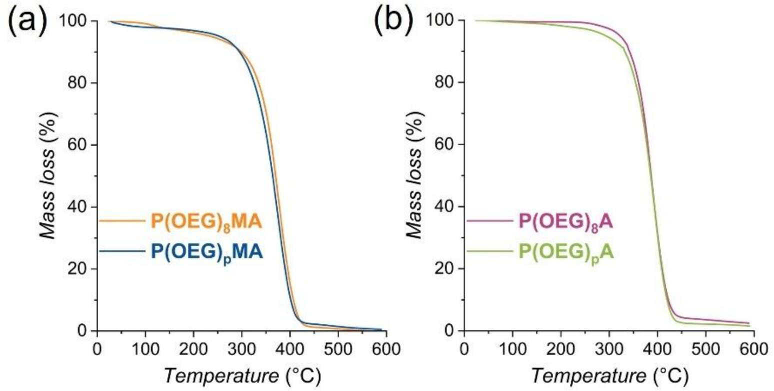

Figure S24. Thermogravimetric analysis (TGA) of polymethacrylates and polyacrylates with discrete and disperse OEG side chains. P(OEG)$_p$MA and P(OEG)$_p$A are obtained from the respective macromonomers with reported molar mass of ~500 and ~480 g mol^{-1}.

Table S10. Activation energy and pre-exponential factor for Li ion transport in PEs with discrete and disperse OEG side chains, obtained from EIS data by means of the VTF equation[a].

Polymer [b]	E_a (kJ mol^{-1})	A (S cm^{-1})
P(OEG)$_8$MA	9.89 ± 0.16	0.29 ± 0.04
P(OEG)$_p$MA	10.07 ± 0.06	0.61 ± 0.03
P(OEG)$_{mp}$MA	8.78 ± 0.17	0.32 ± 0.04
P(OEG)$_8$A	9.44 ± 0.07	0.56 ± 0.03
P(OEG)$_p$A	9.04 ± 0.11	0.46 ± 0.04

[a] The Vogel temperature T_0 was set to $T_0 = T_g - 50$ K.
[b] All polymers were mixed with LiTFSI, at $r = 0.08$.

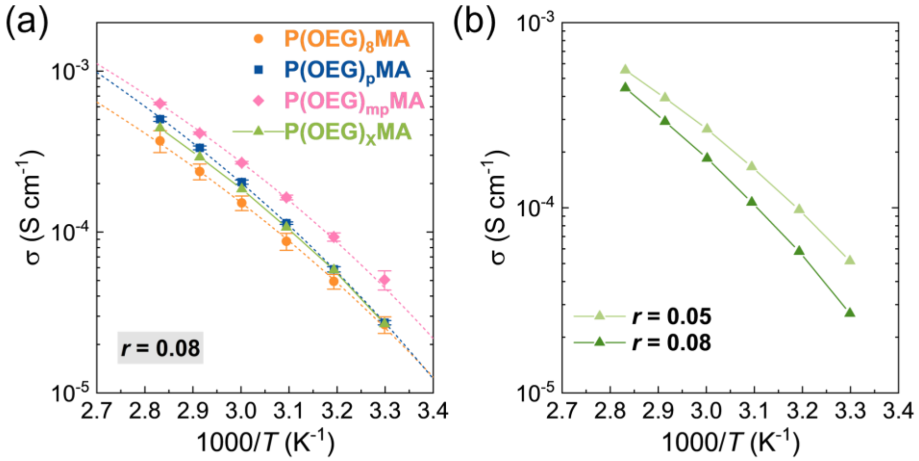

Figure S25. Variation of Li-ion conductivity (measured by EIS) for a PE comprising P(OEG)$_X$MA (75 mol% (OEG)$_p$MA-300, 23 mol% (OEG)$_p$MA-950, 2 mol% (OEG)$_p$MA-500) and LiTFSI, (a) in comparison with other P(OEG)MAs (as in Figure 3) at $r = 0.08$, and (b) as a function of the amount of LiTFSI (expressed as $r = [Li^+]/[EO]$).

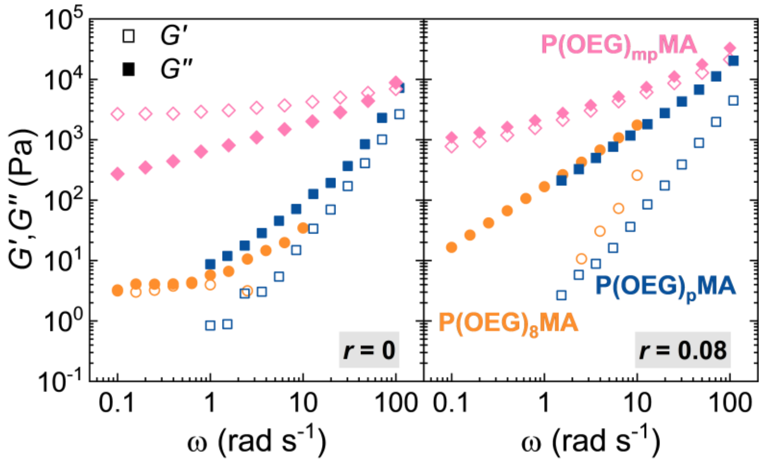

Figure S26. Oscillatory rheology experiments giving the storage (G', empty markers) and loss (G'', filled markers) moduli of polymethacrylates with discrete and disperse OEG side chains, without and with added LiTFSI ($r = 0.08$).

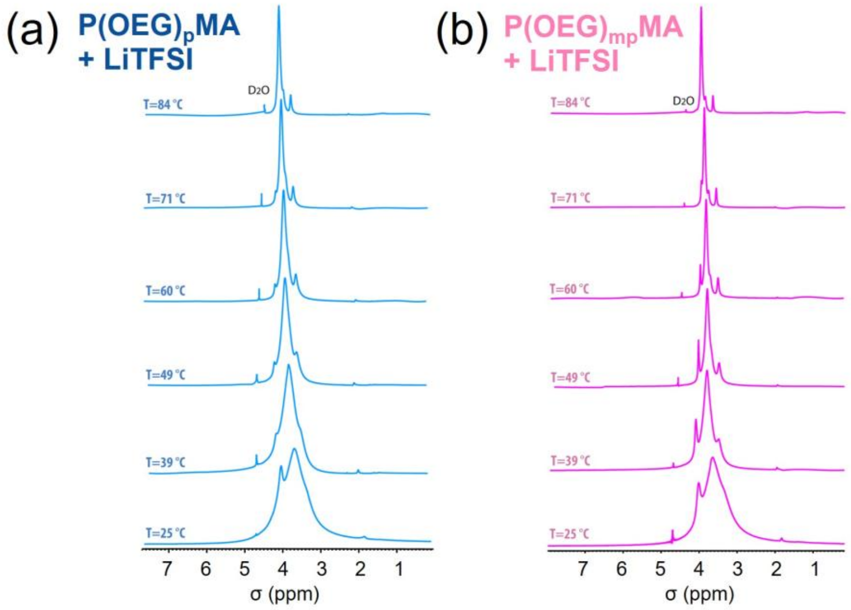

Figure S27. ^{1}H HR NMR spectra of P(OEG)$_p$MA and P(OEG)$_{mp}$MA blended with LiTFSI (r = 0.08) as a function of temperature, in the range of 25-84 °C.

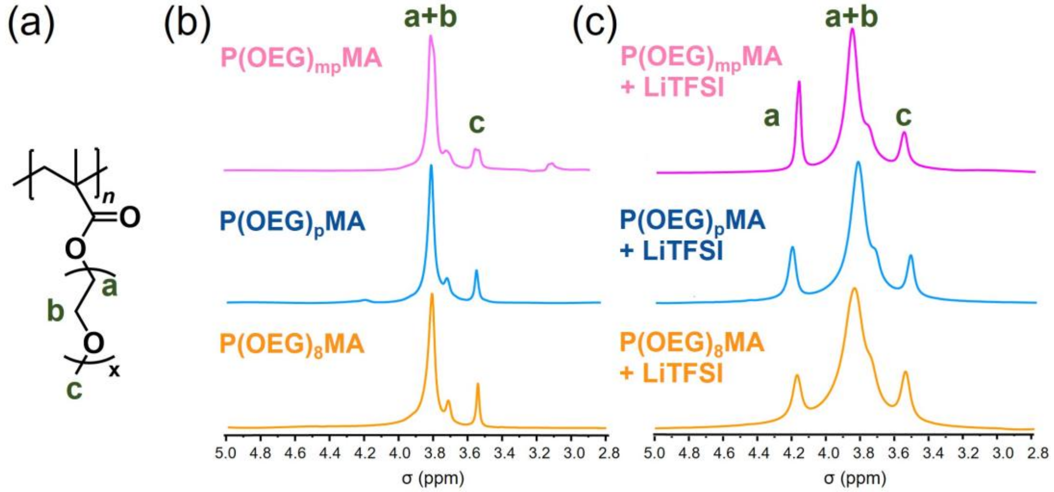

Figure S28. ^{1}H HR MAS NMR spectra of P(OEG)MAs (general structure and proton assignment in (a)), without (b) and with (c) LiTFSI (r = 0.08) recorded at T = 25 °C.

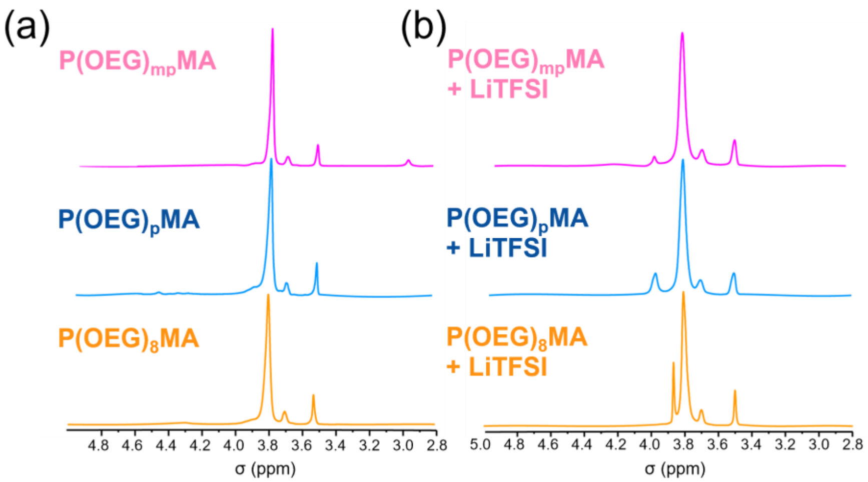

Figure S29. ^{1}H HR MAS NMR spectra of P(OEG)MAs without (a) and with (b) LiTFSI (r = 0.08) recorded at T = 25 °C.

Table S11. T_1 relaxation times of polymethacrylates with discrete and disperse OEG side chains, in the absence and presence of added LiTFSI (r = 0.08), obtained from NMR at T = 25 °C.

	**P(OEG)$_8$MA**		**P(OEG)$_p$MA**		**P(OEG)$_{mp}$MA**	
**Protons** [a]	T_1 **(ms)**	$T_{1,\mathrm{LiTFSI}}$ **(ms)**	T_1 **(ms)**	$T_{1,\mathrm{LiTFSI}}$ **(ms)**	T_1 **(ms)**	$T_{1,\mathrm{LiTFSI}}$ **(ms)**
a		520.3		537.1		474.5
a+b	595.1	622.9	597.7	620.7	527.5	587.3
	674.2		673.9		569.5	
c	675.7	624.1	683.9	630.9	415.2	579.5

[a] ^{1}H NMR signal assignment is shown in **Figure S28**.

Table S12. Self-diffusion coefficients of polymethacrylates with discrete and disperse OEG side chains, in the absence and presence of added LiTFSI (r = 0.08), obtained from PGSE NMR at T = 25 °C.

**P(OEG)$_8$MA**		**P(OEG)$_p$MA**		**P(OEG)$_{mp}$MA**	
D **(10^{-12} m^2 s^{-1})**	D_{LiTFSI} **(10^{-12} m^2 s^{-1})**	D **(10^{-12} m^2 s^{-1})**	D_{LiTFSI} **(10^{-12} m^2 s^{-1})**	D **(10^{-12} m^2 s^{-1})**	D_{LiTFSI} **(10^{-12} m^2 s^{-1})**
4.5	1.3	0.9	1.2	2.7	1.4

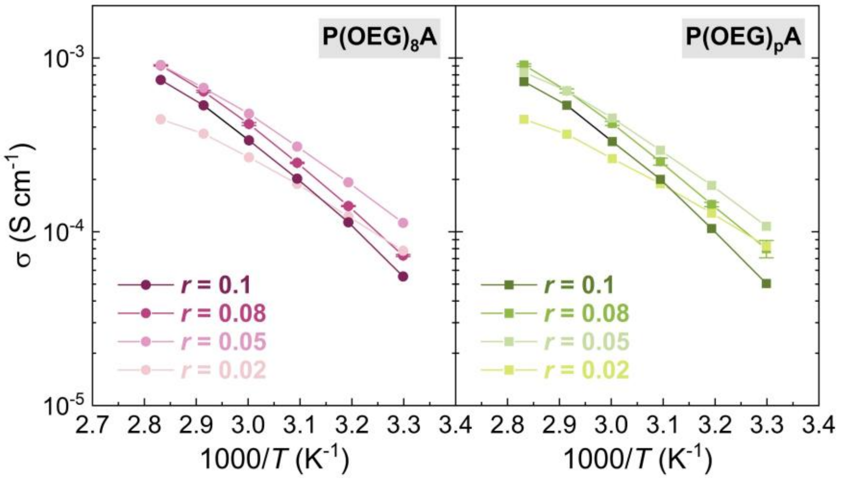

Figure S30. Variation of Li-ion conductivity (measured by EIS) with the amount of LiTFSI (expressed as $r = [Li^+]/[EO]$) for polymer electrolytes based on polyacrylates with discrete (8 EO units) and disperse OEG side chains.

Table S13. Bulk resistance data measured by EIS for POEG(M)A-based electrolytes with discrete and disperse OEG side chains

**P(OEG)8MA**				
		R (Ω)		
T (°C)	r = 0.1	r = 0.08	r = 0.05	r = 0.02
80	60.3	55.1 ± 9.1	48.8 ± 2.7	86.4
70	85.8	84.5 ± 9.8	68.0 ± 3.7	116.3
60	139.5	132.3 ± 13.9	99.7 ± 4.2	162.8
50	266.5	229.6 ± 27.9	154.2 ± 7.7	239.6
40	462.4	406.4 ± 42.6	253.3 ± 14.5	399.8
30	1276.6	765.5 ± 136.4	449.7 ± 30.4	626.2
**P(OEG)pMA**				
		R (Ω)		
T (°C)	r = 0.1	r = 0.08	r = 0.05	r = 0.02
80	63.1	39.6 ± 1.2	38.2 ± 2.3	95.6
70	92.0	59.7 ± 1.6	53.0 ± 3.2	123.5
60	142.5	97.5 ± 2.9	78.5 ± 5.0	174.8
50	247.8	175.4 ± 3.8	117.6 ± 1.3	253.0
40	455.0	339.8 ± 12.9	210.8 ± 11.4	576.1
30	877.3	729.2 ± 22.8	378.9 ± 14.0	896.3

(Continued on next page)

Table S13 (continued)

**P(OEG)$_p$MA**				
		R (Ω)		
T (°C)	r = 0.1	r = 0.08	r = 0.05	r = 0.02
80	45.7	31.7 ± 0.6	35.9 ± 4.3	64.0
70	66.8	48.4 ± 1.0	47.9 ± 4.1	85.3
60	113.3	73.9 ± 1.4	70.5 ± 5.7	118.9
50	202.4	121.5 ± 4.1	112.8 ± 15.2	175.1
40	377.6	214.2 ± 13.0	191.8 ± 22.1	278.7
30	774.0	399.5 ± 55.0	371.9 ± 2.0	460.3
**P(OEG)$_8$A**				
		R (Ω)		
T (°C)	r = 0.1	r = 0.08	r = 0.05	r = 0.02
80	26.6	22.2	22.0	44.9
70	37.3	31.4	31.5	54.3
60	59.4	48.7	45.7	74.2
50	98.6	80.6	74.3	105.7
40	175.7	142.5	103.4	162.7
30	360.0	269.2	177.0	256.2
**P(OEG)$_p$A**				
	R (Ω)			
T (°C)	r = 0.1	r = 0.08	r = 0.05	r = 0.02
80	27.3	21.8	24.1	44.9
70	37.3	30.7	30.8	54.7
60	60.2	47.2	44.1	75.5
50	99.4	78.9	67.6	105.3
40	190.8	139.4	107.6	155.4
30	395.0	260.4	184.9	240.9

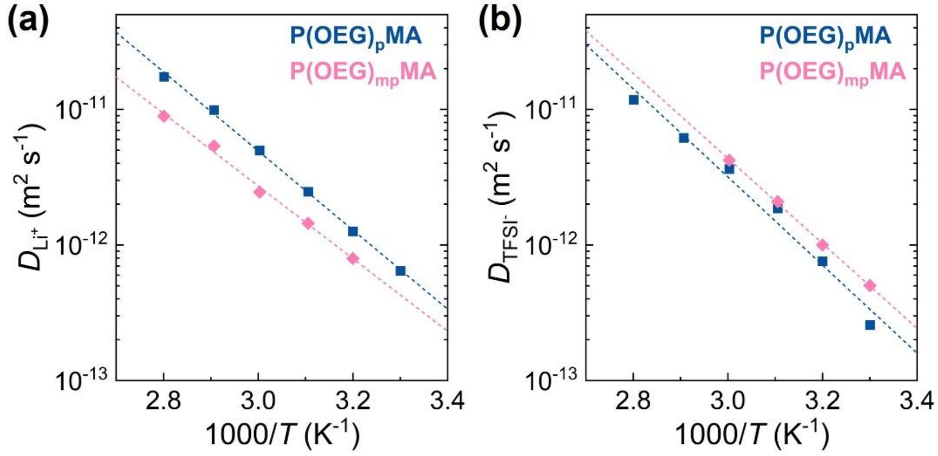

Figure S31. Arrhenius plot for the self-diffusion coefficients of Li^+ (^7Li) and $TFSI^-$(^{19}F) measured by NMR across the temperature range 30-84 °C for P(OEG)$_p$MA and P(OEG)$_{mp}$MA-based electrolytes with r = 0.08.

Table S14. Li-ion conductivity values (σ_{NMR}) calculated from Li^+ diffusion coefficients measured by PGSE NMR, using the Nernst–Einstein equation,[a] for POEGMA-based electrolytes (r = 0.08) with discrete and disperse OEG side chains, and comparison with the corresponding Li-ion conductivities measured by EIS, to estimate the average degree of salt dissociation (α).

	**P(OEG)$_p$MA**			**P(OEG)$_{mp}$MA**		
***T* (°C)**	**σ_{NMR} (10^{-4} S cm^{-1})**	**σ (10^{-4} S cm^{-1})**	**α^b**	**σ_{NMR} (10^{-4} S cm^{-1})**	**σ (10^{-4} S cm^{-1})**	**α^b**
30	0.43	0.27	0.64			
39	0.93	0.59	0.63	0.93	0.93	1.00
49	1.93	1.13	0.59	1.77	1.64	0.93
60	3.70	2.04	0.55	3.22	2.69	0.84
71	6.70	3.33	0.50		4.11	
84	11.7					

[a] Calculated as: $\sigma_{NMR} = \frac{F^2}{RT}\sum c_i z_i D_i$, where c_i is the molar concentration of the ions, z_i is their absolute charge and D_i is their diffusion coefficient, while F is the Faraday constant. [b] Calculated as: $\alpha = \sigma / \sigma_{NMR}$.

References

1. Wu, D.; Chen, A.; Johnson, C. S., An improved diffusion-ordered spectroscopy experiment incorporating bipolar-gradient pulses. *J. Magn. Reson. A* **1995**, *115* (2), 260-264. DOI: 10.1006/jmra.1995.1176.

2. Lorandi, F.; Fantin, M.; Matyjaszewski, K., Atom Transfer Radical Polymerization: A Mechanistic Perspective. *J. Am. Chem. Soc.* **2022**, *144* (34), 15413-15430. DOI: 10.1021/jacs.2c05364.
